# Design, synthesis, and characterization of a novel Zn(II)-2-phenyl benzimidazole framework for the removal of organic dyes

**DOI:** 10.1038/s41598-022-16753-8

**Published:** 2022-07-20

**Authors:** Shabnam Alibakhshi, Ashraf S. Shahvelayati, Shabnam Sheshmani, Maryam Ranjbar, Saeid Souzangarzadeh

**Affiliations:** 1 Department of Chemistry, College of Basic Sciences, Yadegar-e-Imam Khomeini (RAH) Shahre Rey Branch, Islamic Azad University, Tehran, Iran; 2 Department of Chemical Technologies, Iranian Research Organization for Science and Technology (IROST), Tehran, Iran

**Keywords:** Environmental sciences, Chemistry, Materials science, Nanoscience and technology

## Abstract

A novel Zn (II) organic framework comprising 2-phenyl benzimidazole (ZPBIF-1) was synthesized by using a solvothermal method. The characterization of the synthesized MOF was performed utilizing XRD, SEM, FT-IR, ^1^H-NMR, ^13^C-NMR, MS, XPS, TG/DTA, and N_2_ sorption analysis. ZPBIF-1 was successfully utilized to remove Acid red 88, Basic Violet 14, Basic Blue 54, and Congo red dyes in aqueous solutions. In this study, some parameters, including adsorbent dosage, initial dye concentration, contact time, temperature, and pH, were examined. To evaluate the experimental data, Freundlich, Langmuir, Temkin, and Dubinin-Radushkevich isotherm models were used. In this case, Langmuir is the most suitable model. Several kinetic models, including First-order, pseudo-first-order, second-order, and Pseudo-second-order kinetic models, Elovich's, and Weber's intraparticle diffusion models, were utilized to comprehend the detailed adsorption process. According to the pseudo-second-order kinetic model, dye sorption kinetics is best described. In addition, thermodynamic parameters like enthalpy (ΔH°), Gibbs free energy (ΔG°), and entropy (ΔS°) were also achieved and analyzed. The experimental studies thus suggest that Zn (II) metal–organic framework based on 2-phenyl benzimidazole could be a promising candidate for eliminating dyes from aqueous solution. Hence, the experimental studies suggest that a Zn (II) metal–organic framework based on 2-phenylbenzimidazole could be a promising candidate for eliminating dyes from aqueous solution. The maximum adsorption capacity of ZPBIF-1 was 1666.66, 1250, 1000, and 1250 mg/g for Acid red 88, Basic violet 14, Basic blue 54, and Congo red dyes, respectively. Furthermore, this method was used to remove contaminant dyes from textile wastewater, and an acceptable result was obtained.

## Introduction

Colored chemicals are symbolized by dyes in several industries, such as leather, textiles, paper, and plastic. Due to dye consumption and production, as well as difficulty removing dyes during coagulation procedures, colored wastewater is produced directly. In general, most dyes are synthetic and contain aromatic rings^1^, making them carcinogenic and mutagenic, not biodegradable, and inert if discharged into waste streams. Potentially, they can have acute or chronic effects on organisms exposed to them. Sunlight is also absorbed or reflected by water-soluble dyes inhibiting the bacteria growth. Furthermore, they are visible and lead to esthetic pollution even at low concentrations^2^. Thus it is significantly essential to remove the color from aquatic systems^3,4^.

Many methods for removing dyes from wastewater have been developed, including adsorption, chemical precipitation, oxidation, and photocatalytic degradation^5–10^. Among them, the adsorption technique is simple to apply and offers considerable advantages in this regard. Adsorption is a method for removing a wide range of compounds from industrial wastewater. In most cases, adsorption is employed for removing low levels of non-degradable organic compounds from groundwater, drinking water preparation, processing water, or as a tertiary cleanse after biological water treatment. During adsorption, molecules in a liquid bond with those on a solid surface, and since adsorbents have a large surface area, they are suitable for adsorption^11^. In fact, it can be used with almost any dye or dye combination without requiring any pretreatment or equipment. Furthermore, adsorption procedures are economical since they can be performed under favorable conditions, decreasing the actual cost of the adsorbent, which can be chosen accordingly^12,13^. Surface area, short adsorption times, high adsorption capacity, and an environmentally friendly and economically efficient production process is the primary properties of a suitable adsorbent^14,15^.

Recently, as a new class of porous crystalline materials, metal–organic frameworks (MOFs) have been used for a variety of applications such as chemical separation (such as carbon capture as a global challenge), gas (green gases, hydrogen, and methane) storage, catalysis, sensors, drug delivery and storage, membranes, and the removal of hazardous wastes (like heavy metals, dyes, nitrogen, and sulfur-comprising compounds)^16–25^. MOFs with the porous structures are one of the most promising groups of novel materials for adsorbing hazardous compounds from the liquid phases, such as sulfur and nitrogen compounds from non-aqueous and aqueous media^26,27^.

As part of our continuing efforts toward the development of green chemistry and the removal of organic pollutants^28,29^, a novel Zn (II) organic framework based on 2-phenyl benzimidazole (ZPBIF-1) was synthesized and characterized via Fourier transform infrared (FT-IR), scanning electron microscope (SEM), X-ray diffraction (XRD), nuclear magnetic resonated (^1^H-NMR, ^13^C-NMR), mass spectrometry (MS), X-ray photoelectron spectroscopy (XPS), and thermogravimetric differential thermal analyses (TG/DTA).

**Figure 1:**
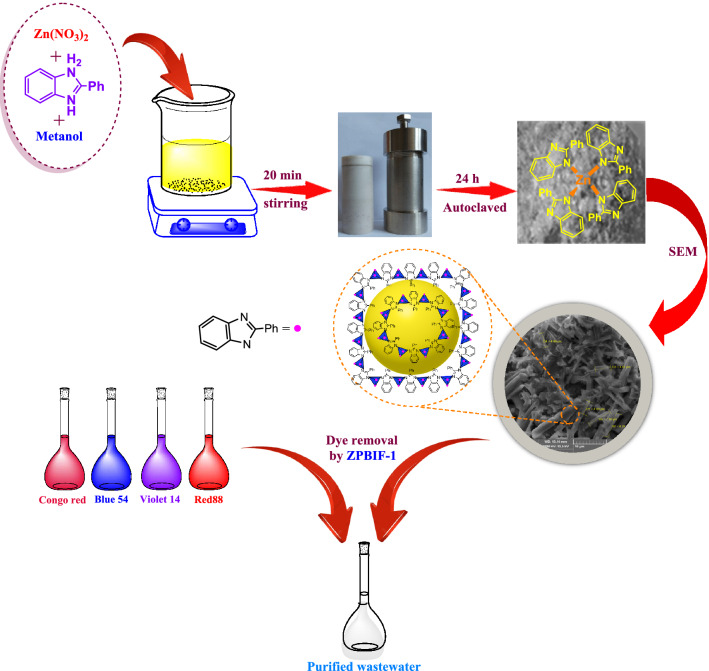
Preparation of ZPBIF-1 metal organic-framework.

The objective of this work was to assess the applicability of novel MOF (ZPBIF-1) for the removal of four different categories of dyes from aqueous solution with a variety of experimental conditions (initial dye concentration temperature, contact time, pH, and adsorbent dose). Extensive studies of isotherms, kinetics, thermodynamics, and equilibrium were carried out to determine this adsorbent's performance on acid red 88, basic Violet 14, basic Blue 54, and Congo red dyes (The structures of dyes are provided in Supplementary file 5). Lastly, the adsorption mechanism of these dyes on the ZPBIF-1 surface was adequately discussed. The high efficiency and simplicity of the ZPBIF-1 preparation method, its effectiveness, reusability, and high adsorption capacity, make its industrial use very promising (Fig. 1).

## Materials and methods

### Materials

Zinc nitrate hexahydrate (Zn(NO_3_)_2_·6H_2_O 98%), methanol, 2-phenyl benzimidazole, sodium hydroxide, hydrochloric acid, and chloroform were purchased from Merck Company. All chemicals were commercially available in analytical grade and were utilized without further purification. All selected dyes including Acid red 88 (C. I. No: 1658-56-6), Basic Violet 14 (C. I. No: 632-99-5). Basic Blue 54 (C. I. No: 15000-59-6) and Congo red (CR) (C. I. No: 573-58-0) were obtained from Nordex international, D.Z.E Dye Company in the UK. The natural textile wastewater, including orange and red Bemacron SER-DL, was collected from the Farizad Textile Dying Factory in Varamin, Iran.

### Apparatus

The FT–IR spectra were recorded on the Tensor 27 Burker spectrophotometer in 500–4000/cm. ^1^H NMR and ^13^C NMR spectra recorded on a on Bruker 250 MHz spectrometer with DMSO-d_6_ as a solvent. The crystalline nature of ZPBIF-1 was analyzed by X-ray diffraction at room temperature with Co Kα radiation (model X’Pert PRO MPD, Panalytical, Made in the Netherland). The X-ray photoelectron spectroscopy (XPS) was conducted on the Bes Tec (USA) using Mg radiation. A Netzsch Thermoanalyze (Mettler Toledo, Swiss) was used for thermogravimetric analysis (TGA) with a heating rate of 50 ml/min in air. A combination of electrospray ionization mass spectrometry (ESI–MS) (5975C, Agilent Technologies, USA) was applied. The morphologies and elemental analysis were assessed by a scanning electron microscope SEM (Mira III, TeScan, Czech Republic) supplied with energy dispersive X-ray analysis (EDXA). The concentration level of remaining COD and BOD in wastewater sample was determined by the Standard Methods, using a USA-HACH spectrophotometer and Lutron DO meter. The UV–Vis spectrophotometer (Cary 60, Agilent Technologies USA) was utilized to determine the concentrations of the organic dyes at wavelengths of 497, 583, 497, and 559 nm, respectively. The specific surface area of samples was determined with Brunauer–Emmett–Teller (BET) method using the adsorption–desorption isotherms at 77 K (BEL, Belsorp mini II, Made in Japan).

### Preparation of ZPBIF-1

Typically, A mixture of zinc nitrate hexahydrate (Zn(NO_3_)_2_·6H_2_O) (3.02 mmol, 0.9 g) and 2-phenyl benzimidazole (1.54 mmol, 0.3 g) was dissolved in methanol (35 mL), then autoclaved. Teflon-lined stainless steel (autoclave) was heated for 24 h at 90 °C in a programmable oven and cooled at room temperature at 0.4 °C/min. After the addition of chloroform to yellow liquid, the colorless polyhedral crystals of ZPBIF-1 were formed that were washed with methanol, then dried under vacuum for 6 h at 50 °C to give the desired product in 95% yield.

### Adsorption experiments

The optimal conditions and influencing parameters for dye removal by ZPBIF-1 were investigated in the adsorption procedure. The Optimum adsorbent quantity was determined by combining different amounts of ZPBIF-1 (0.001–0.01 g) with dye solution (10 mL) for 20 min at 25 °C, with an initial dye concentration of 20 mg/L, and pH = 2–8 under continuous stirring at 400 rpm. A pH adjustment was achieved by adding sodium hydroxide (0.1 N) or hydrochloric acid (0.1 N) to the dye solution before combining it with the adsorbent. The effects of initial dye concentration (10–800 mg/L) were investigated using 0.008 g adsorbent at pH = 2 and 25–65 °C for 20 min. UV–Vis spectrophotometers were used to measure dye solution concentrations and adsorption. The dye solution concentration followed by adsorption was analyzed employing a UV–Vis spectrophotometer. Afterward, the amounts of dye adsorbed at different times (qt, mg/g) and dyes' removal efficiency were determined as follows:

1 $$q_{t} = \frac{{(C_{o} - C_{t} ) \times V}}{m}$$

2 $$removal\;\% = \frac{{C_{o} - C_{e} }}{{C_{o} }}$$

which, C_e_, C_0_, and C_t_ (mg/L) were the equilibrium concentration, initial concentration, and concentration at different times, respectively. V was the volume of dye solution, and m (g) was the mass of ZPBIF-1. As part of the kinetic studies and optimization of adsorption capacity, the adsorbent dosage (0.01, 0.02, 0.06, 0.08, and 1 g/L) and dyes concentration (10–800 mg/L) were selected. The same procedure was repeated at 308, 318, and 338 K to assess the effects of temperature on dye adsorption.

### Recyclability and stability study

The Efficiency of ZPBIF-1 as a selective dye adsorbent from waste water can be leveraged by its superior recyclability. Recycling was performed on ZPBIF-1 using optimized conditions such as dose of 0.08 g/L, dye concentration of 20 mg/L, pH 2, contact time of 20 min, and agitation rate of 400 rpm. The major recycling procedure can be described as follows: to organic dye-loaded ZPBIF-1 (0.008 g) from the first adsorption experiment, NaOH 0.1 M (10 mL) and ethanol %50 (10 mL) were added and mixed for 20 min. Following filtration and washing with H_2_O/EtOH, samples were dried and reactivated at 50 °C under vacuum for 60 min and used for reusability studies. UV–Vis spectroscopy was used to check for residual dye traces.

## Results and discussion

### Characterization of ZPBIF-1

A new zinc metal–organic framework, Tetrakis (2-phenylbenzoimidazol-1-yl) zinc (ZPBIF-1), was synthesized by autoclaving 2-phenyl benzimidazole and Zn (NO_3_)_2_ in methanol. The structure of ZPBIF-1 was characterized by FT-IR, ^1^H-NMR, ^13^C-NMR, and mass spectral data. The FT-IR spectrum of ZPBIF-1 exhibited the characteristic peaks of Zn moieties at 600/cm and aromatic moieties at 620–826/cm. Also, the stretch absorptions of C=C aromatic appeared in pairs at 1633/cm and 1468/cm. The adsorption band in the regions 1458, 1669, and 759, 1227, 1388 can be assigned to the C–N, C=C stretching and bending vibration, respectively.

Due to the symmetry of this compound, ^1^H-NMR and ^13^C-NMR only revealed peaks for one of the four ligands. Therefore, the ^1^H NMR spectrum of ZPBIF in DMSO showed two doublet-doublet signals (AX system) in δ = 7.51 and 7.80 ppm for CHs-aromatic of benzimidazole ring along with characteristic signals for phenyl group at 2-position of imidazole moiety. The proton-decoupled ^13^C-NMR spectrum of ZPBIF-1 in DMSO showed 8 distinct signals related to 28 CH and 24 C in agreement with the proposed structure. It showed one distinct resonance for the N–C=N group. Partial assignments of aromatic and imidazole resonances were given in the experimental report.

C_52_H_36_N_8_Zn (838.30), Colorless crystals, yield: 94.76%. IR (KBr) (νmax/cm): 3107, 3062, 1633, 1481, 1388, 1227, 759, 697/cm. ^1^H NMR (DMSO): δ = 7.51 (8 H, dd, ^*3*^*J* = 6.0, ^*4*^*J* = 3.1, 8 CH), 7.70–7.73 (12 H, m, 12 CH_o,p_-Ph), 7.80 (8 H, dd, ^*3*^*J* = 6.0, ^*4*^* J* = 3.1, 8 CH), 8.17–8.20 (8 H, m, 8 CH_m_-Ph) ppm. ^13^C NMR (DMSO): *δ* 114.3 (8 C), 124.7 (4 C), 125.2 (8 CH), 127.6 (8 CH_o_-Ph), 129.6 (8 CH_m_-Ph), 132.1 (4 CH_p_-Ph), 133.5 (8 C), 149.6 (4N–C=N) ppm.

The mass spectra of compound represented molecular ion peak at proper *m/z* value and the base peak appeared at *m/z* 194. EI-MS: m/z (%) = 836 (M+, 5), 194 (100), 193 (30), 116 (10), 104 (7), 91 (8), 90 (13), 77 (15). The IR, ^1^HNMR, ^13^CNMR, and Mass spectra for Zn (II)-2-phenyl benzimidazole framework (ZPBIF-1) are provided in the Supplementry file 5.

**Figure 2:**
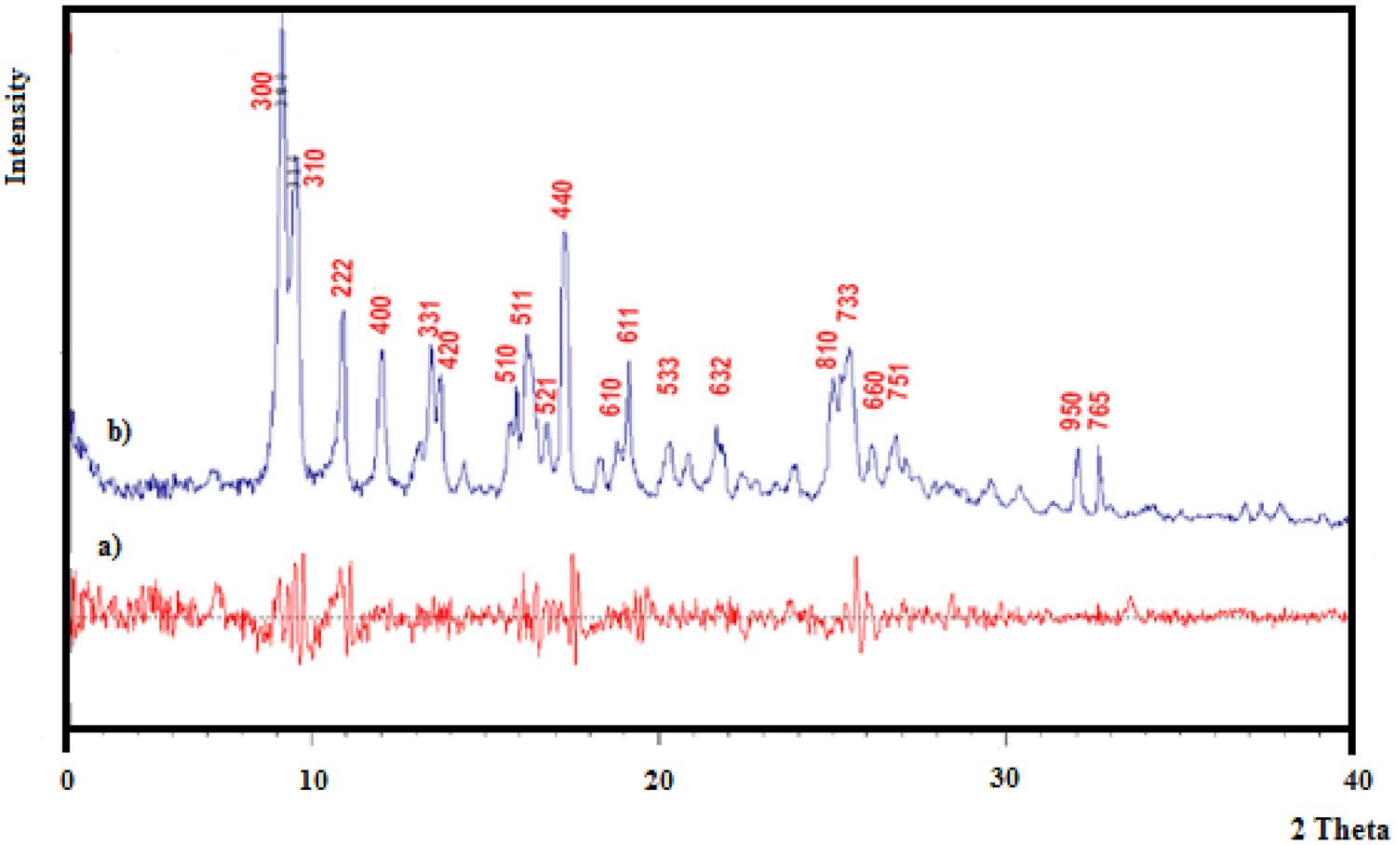
(**a**) The simulated PXRD pattern of ZIF-11, (**b**) XRD pattern of Zn (II)-2-phenyl benzimidazole framework (ZPBIF-1). The raw data for this figure is provided in the Supplementary file 1.

According to the diffraction pattern and comparison with similar composition ZIF-11standard card, it concludes ZPBIF-1 crystals with ideal inner structures had been successfully constructed. The simulated PXRD pattern of ZIF-11 and XRD pattern of Zn(II)-2-phenyl benzimidazole were shown in Fig. 2a,b, respectively. In the Fig. 2b pattern, the crystalline nature of the MOF structure crystallized at cubic crystal system was obtained at 2θ = 9.14, 9.51, 10.86, 11.99, 13.43, 13.69, 15.88, 16.24, 16.76, 17.27, 18.80, 19.10, 20.24, 21.62, 24.99, 25.41, 26.11, 26.75, 32.01, 32.64º at (003), (013), (222), (004), (133), (024), (015), (115), (125), (044), (016), (116), (335), (236), (018), (337), (066), (157), (059), and (567) miller indices, respectively^30,31^.

**Figure 3:**
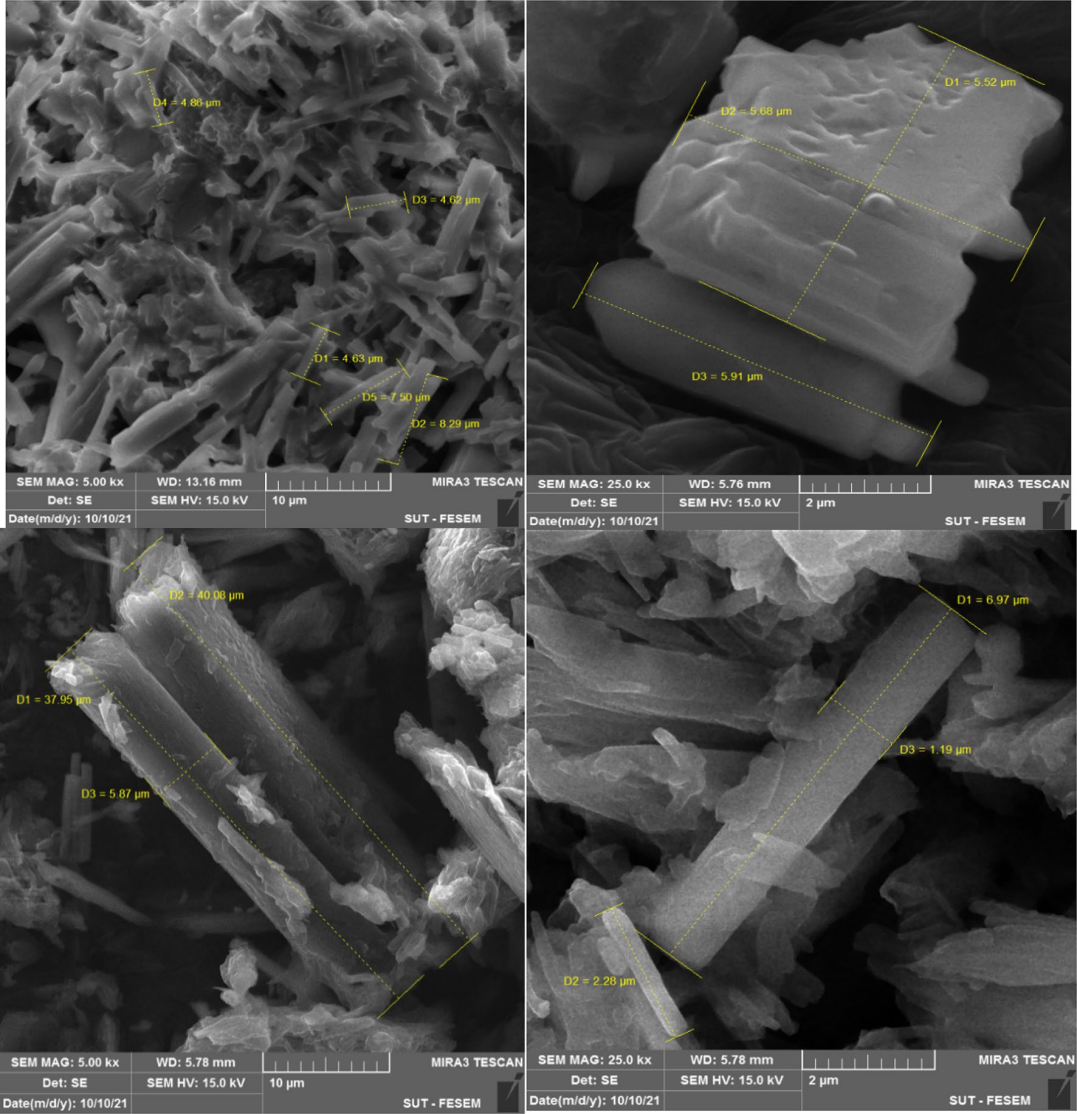
Scanning electron microscopy images of ZPBIF-1.

**Figure 4:**
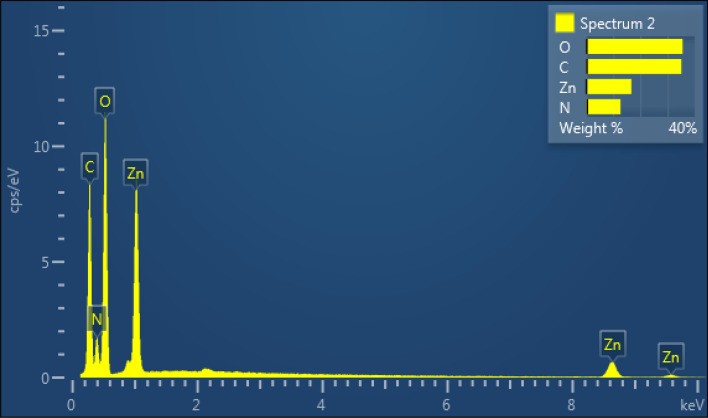
Energy-dispersive X-ray analysis of ZPBI-MOF. The raw data for this figure is provided in the Supplementary file 1.

Metal–organic frameworks (MOFs) are hybrid materials comprising organic linkers and metal ions with high porosity and crystallinity. To study the morphologies, SEM images of the ZPBIF-1, investigated at the high magnifications, show regular rods with the hexagonal face (Fig. 3). Furthermore, it suggests the formation of ZPBIF-1 with homogeneous distribution throughout the MOF. The average size of 40.08 ± 4.62 µm, Energy-dispersive X-ray spectroscopy (EDAX) analyses (Fig. 4) of the ZPBI-MOF show C, O, N, and Zn elements 35.06, 12.69, 35.56, 16.69, respectively.

**Figure 5:**
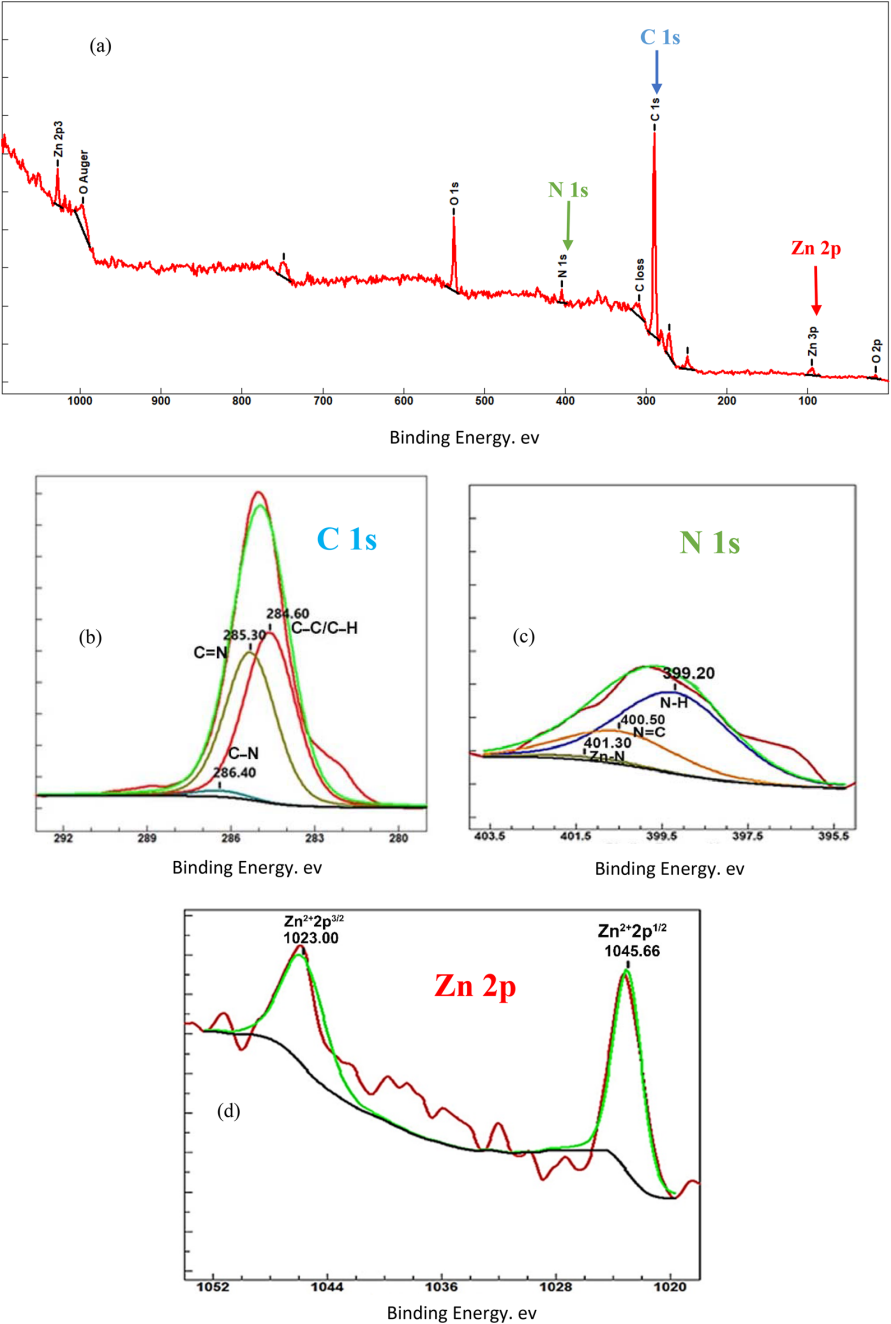
XPS spectra of ZPBI-MOF with full survey (**a**), C 1 s (**b**), N 1 s (**c**), and Zn 2p (**d**). The raw data for this figure is provided in the Supplementary file 3.

A XPS spectrum of the new MOF was prepared in order to determine its oxidation states and chemical bonds. According to Fig. 5a, the presence of constituted elements (N 1 s, C 1 s, and Zn 2p) was proved. As seen in Fig. 5b, high-resolution XPS of the C 1 s spectrum was explained by three well-fitted curves, as well as their respective binding energies. C–C/C–H (saturated carbons), C=N (unsaturated ring), and C–N bonds cause prominent peaks at 284.60, 285.30, and 286.40 eV. Figure 5c confirms the existence of coordination Zn–N bonds at 401.30 eV. N=C and N–H may be responsible for other curves at 400.50 and 399.29 eV, respectively^32,33^. Ultimately, the oxidation states of zinc elements: Zn^2+^ 2p^1/2^ and Zn^2+^ 2p^3/2^ could be proved at notable peaks at 1045.66 and 1023 eV, respectively (Fig. 5d).

**Figure 6:**
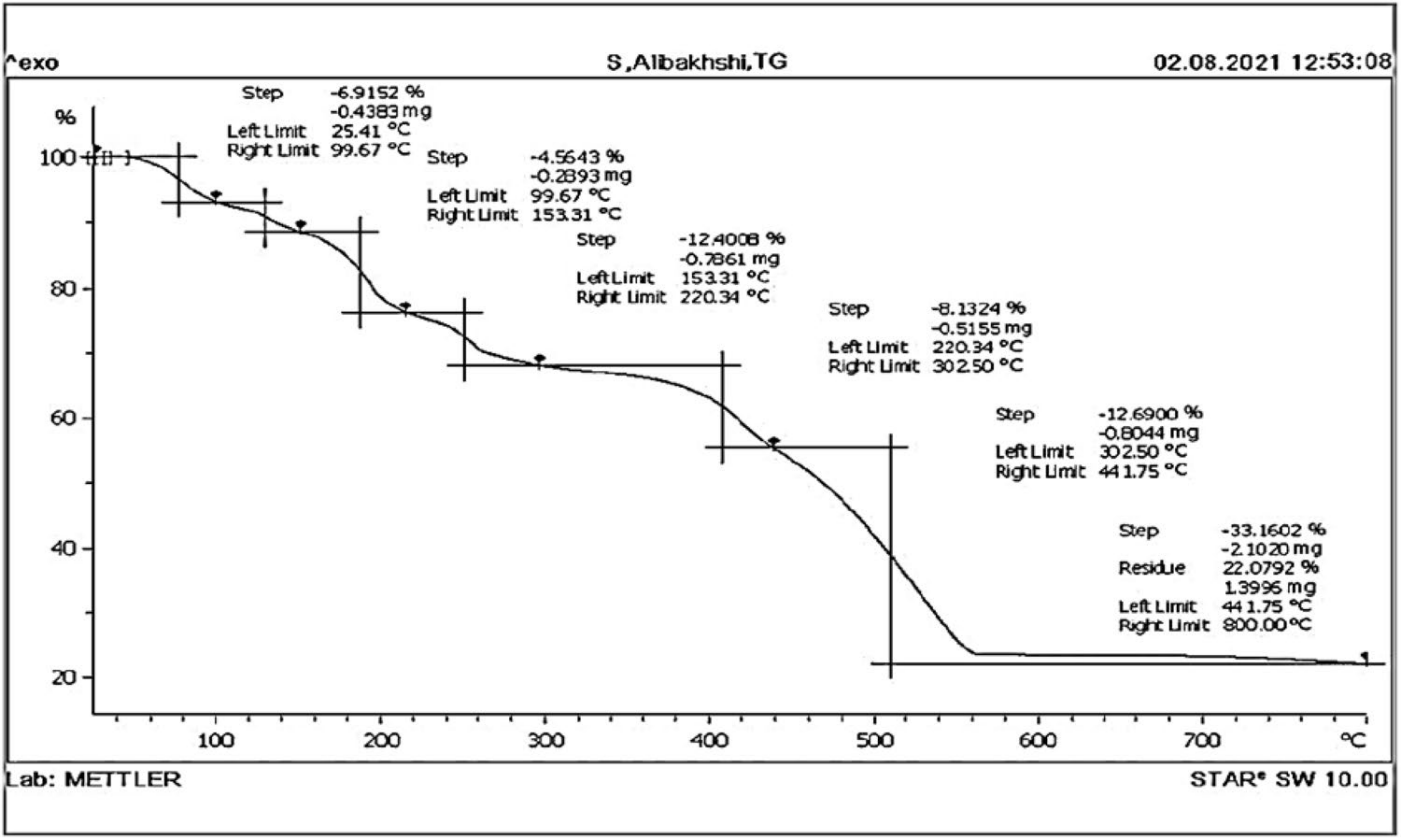
TGA of ZPBIF-1. The raw data for this figure is provided in the Supplementary file 4.

To investigate the thermal stability of the prepared catalyst, derivative TG (DTG) and thermogravimetric analysis (TGA) were carried out. Thermal degradation assessments of solid materials are essential since several applications are based on their thermal stability. The result of the most significant property in the TGA for the ZPBIF-1 was that slight weight loss was found between the temperature range of 100–230 °C, revealing that the ZPBIF-1 was stable up to 400 °C (Fig. 6).

It was indicated that a sharp weight-loss step of over 33.1602% was found by incrementing the temperature from 302.50 to 800 °C. This reveals the thermal decomposition of the ZPBIF-1 in that temperature range. This result of TGA confirmed the applicability of the ZPBIF-1 across various temperatures. The TG analysis of ZPBIF-1 revealed that the MOF was almost constant up to 400 °C with a bit of mass loss (12%). Then a fast and vast weight loss happened from 302.50 to 800 °C, probably due to the exothermic decomposition of ligand (2-phenyl benzimidazole) in these temperatures.

N_2_ adsorption–desorption analysis was employed for obtaining total pore volume (V_m_) and specific surface area of sample. Langmuir surface area and V_m_ of the ZPBIF-1 were 370.73 m^2^/g and 85.176 cm^3^/g, respectively (The related diagram has been shown in Supplementary file 6).

### Adsorption studies

Dye adsorption of Acid Red 88, Basic Violet 14, Basic Blue 54, and Congo red was investigated using ZPBIF-1. Initial dye concentration (20 mg/L) as a function of time (10–60 min) using ZPBIF-1 (0.08 g/L) was studied. It was clear that the ZPBIF-1 was capable of removing 51.7% Acid red 88, 91.7%, Basic violet 14, 99% Basic blue 54, and 97.68% Congo red within 10 min. The removal rate reached 100% within 20 min contact time.

### Effect of pH on dye adsorption

**Figure 7:**
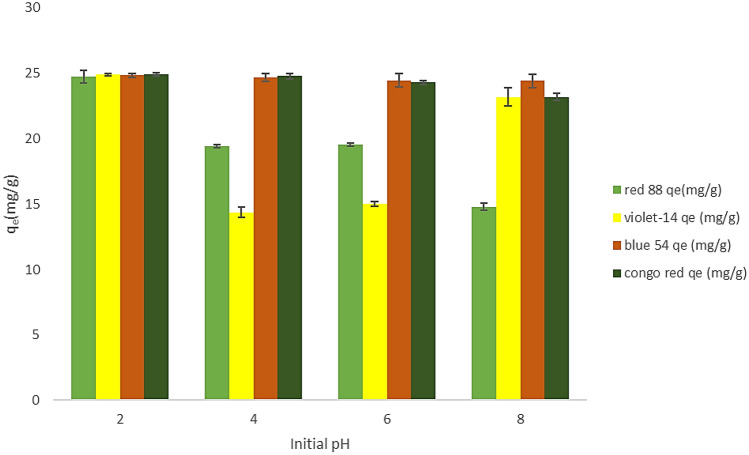
Effect of pH value on dye adsorption (Initial dye concentration 20 mg/L, Adsorbent mass 0.08 g/L, contact time 20 min, Tem. 25 °C, Stirring 400 rpm). The raw data for this figure is provided in the Supplementary file 1.

In dye adsorption, pH is crucial since it determines the degree of ionization of the adsorptive molecules and the surface charge of the adsorbent. The impact of initial solution pH on the adsorption capacity of dyes onto the ZPBIF-1 was recognized at various levels (pH = 2–8) at fixed values of critical parameters (adsorbent dosage 0.008 g/10 mL, dye concentration 20 mg/L, and contact time 20 min). According to Fig. 7, the highest adsorption capacity occurred at pH = 2 for all the dyes containing Acid red 88, Basic violet 14, Basic blue 54, and Congo red. As the solution pH increases from 2 to 8, the adsorption capacity q_t_ (mg/g) decreases for Acid red 88 (an anionic dye). For the other dyes, the pH increase does not significantly reduce the adsorption capacity. This may be due to a large number of functional groups in dyes and the different interactions such as hydrogen bonding, dipole–dipole forces between dye and adsorbent^34,35^.

### Effects of contact time

The effect of contact time on ZPBIF-1 adsorption capacity and dye removal percentage was evaluated under optimal conditions (adsorbent dosage 0.08 g/L, pH = 2, initial dye concentration 20 mg/L, and 25 °C). In this study, the removal efficiency of all dyes was determined as a function of contact time ranging from 10 to 60 min. Based on the results the percent removal and adsorption capacity of the ZPBIF-1 first increase significantly, then increment at a relatively slow speed with the contact time until equilibrium is reached. The most significant adsorption efficiency was obtained after 20 min of dye contact with ZPBIF-1. It is ascribed to the fact that many vacant surfaces are accessible for adsorption at the beginning. After a while, it becomes harder and harder to find the remaining vacant active sites. Hence, in this study, 20 min was selected as the equilibrium contact time.

### Effect of ZPBIF dosage

**Figure 8:**
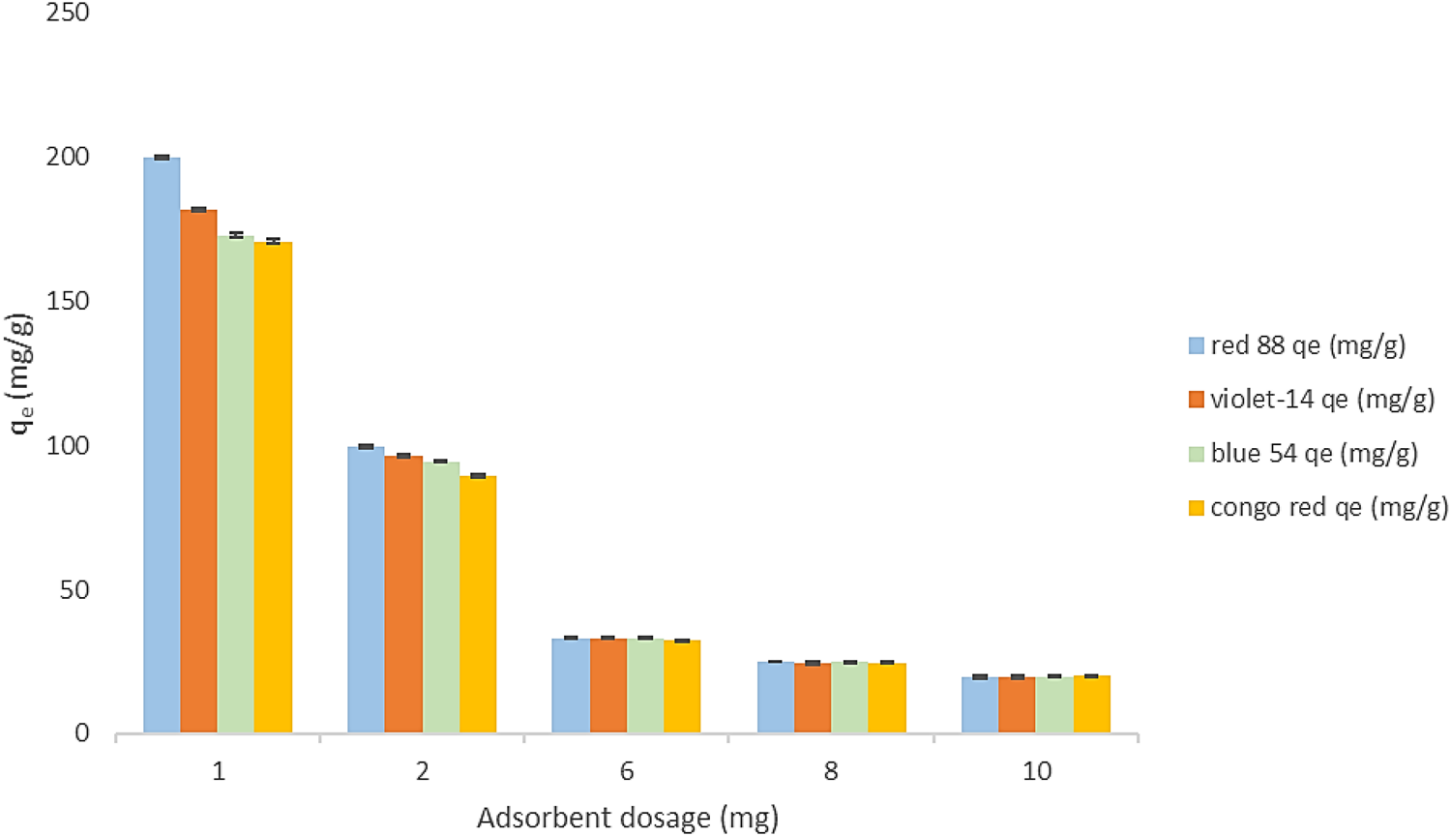
Effect of adsorbent dose (1–10 mg) on dye adsorption (Initial dye concentration 20 mg/l, Adsorbent mass 0.08 g/L, Contact time 20 min, Tem. 25–65 °C). The raw data for this figure is provided in the Supplementary file 1.

Adsorbent concentration is an essential parameter because it indicates the most adsorbent capacity for an initial dye concentration. The optimal amount of ZPBIF-1 to adsorb dyes was determined by adsorption tests performed using 0.001–1 g/L adsorbent at pH = 2, 25 °C, and initial dye concentration (20 mg/L) for 20 min. Figure 8 shows that 100, 91, 86.8, and 85.87% of Acid red 88, Basic violet 14, Basic blue 54, and Congo red were removed from their aqueous solutions when 0.001 g ZPBIF-1 was used as an initial dosage. The best dosage of ZPBIF-1 found to remove Congo red was 0.008 g, while the dosages for Acid red 88, Basic violet 14, and Basic blue 54 were 0.001, 0.006, and 0.006 g, respectively. By increasing the dosage of ZPBIF-1 by 0.008 g, the removal percentage of all dyes increased and ultimately reached 100% for each dye. Further increase of the adsorbent dosage did not influence the removal of dyes, so the dosage of 0.008 g was selected. Additionally, Fig. 8 discusses the removal rate (R%), which increases with incrementing adsorbent amount until achieving equilibrium. This can be explained by an increase in the adsorbent's specific surface area and the availability of more active sites to adsorb dye. In this study, the removal rate of dyes was 100% at 0.008 g of ZPBIF-1, which was considered the optimal adsorbent dose.

### Effect of temperature

**Figure 9:**
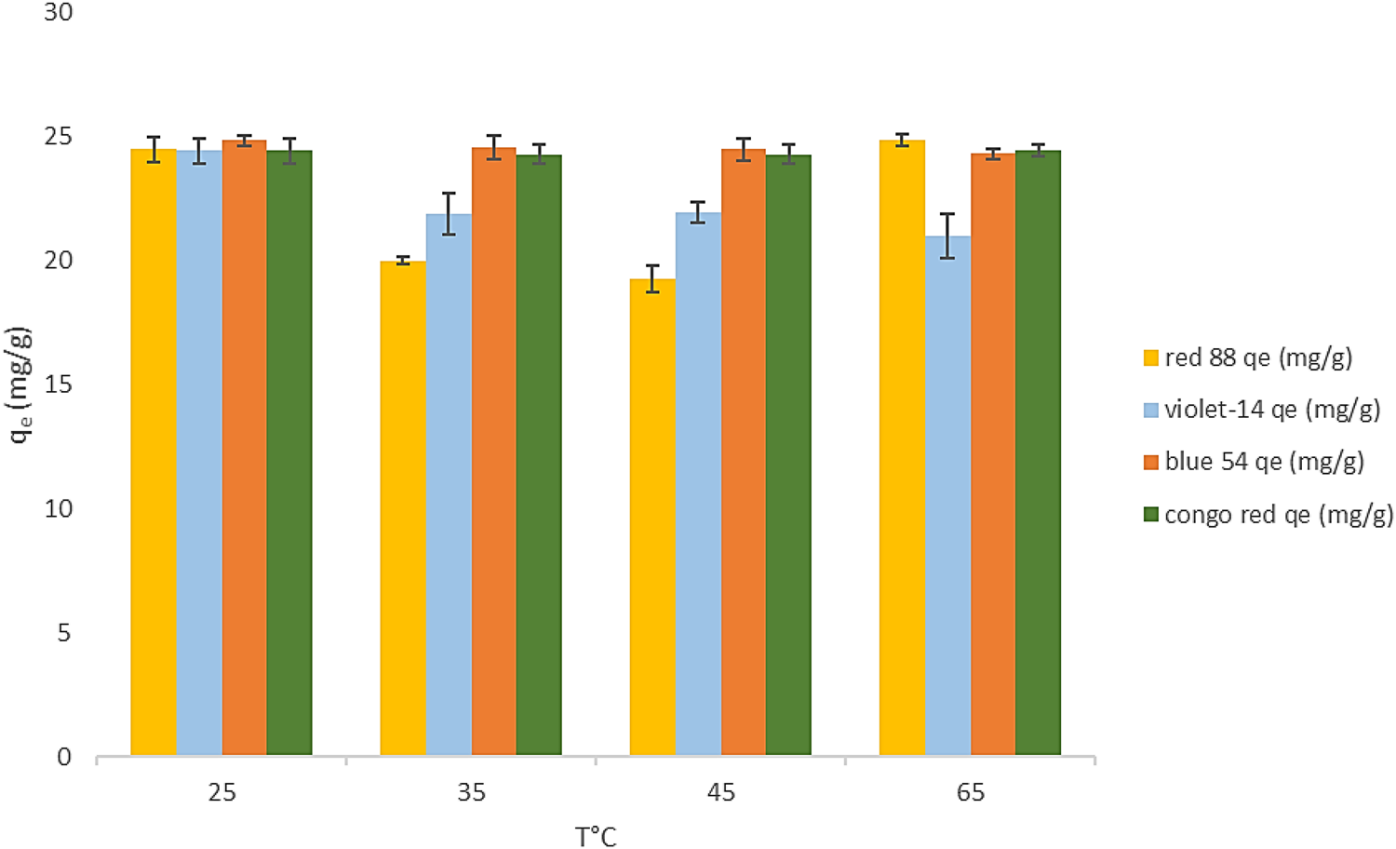
Effect of temperature (25–65 °C) on dye adsorption (Initial dye concentration 20 mg/L, Adsorbent mass 0.08 g/L, contact time 20 min, Stirring 400 rpm). The raw data for this figure is provided in the Supplementary file 1.

The temperature is the main adsorption parameter that could play an essential role in the adsorption process. The temperature severely affects the solution viscosity and thus the adsorption capacity. Therefore, it is necessary to determine the optimum temperature for the adsorption procedure. At the initial concentration of dye 20 mg/L, the effects of temperature on the adsorption of ZPBIF-1 were investigated under optimal conditions (pH = 2, initial dyes concentrations 20 mg/L, stirring time 20 min). Figure 9 showed removal efficiencies for Acid red 88, Basic violet 14, Basic blue 54, and Congo red as a function of temperature ranging between 298 and 323 K. Also, the effect of temperature (298, 273,313, and 323 K) on the adsorption capacity of dye was represented. It is evident that the increase in temperature negatively impacted the removal of dyes. The optimal temperature for the removal of dyes by the ZPBIF-1 was 25 °C.

### Effects of initial dye concentration

**Figure 10:**
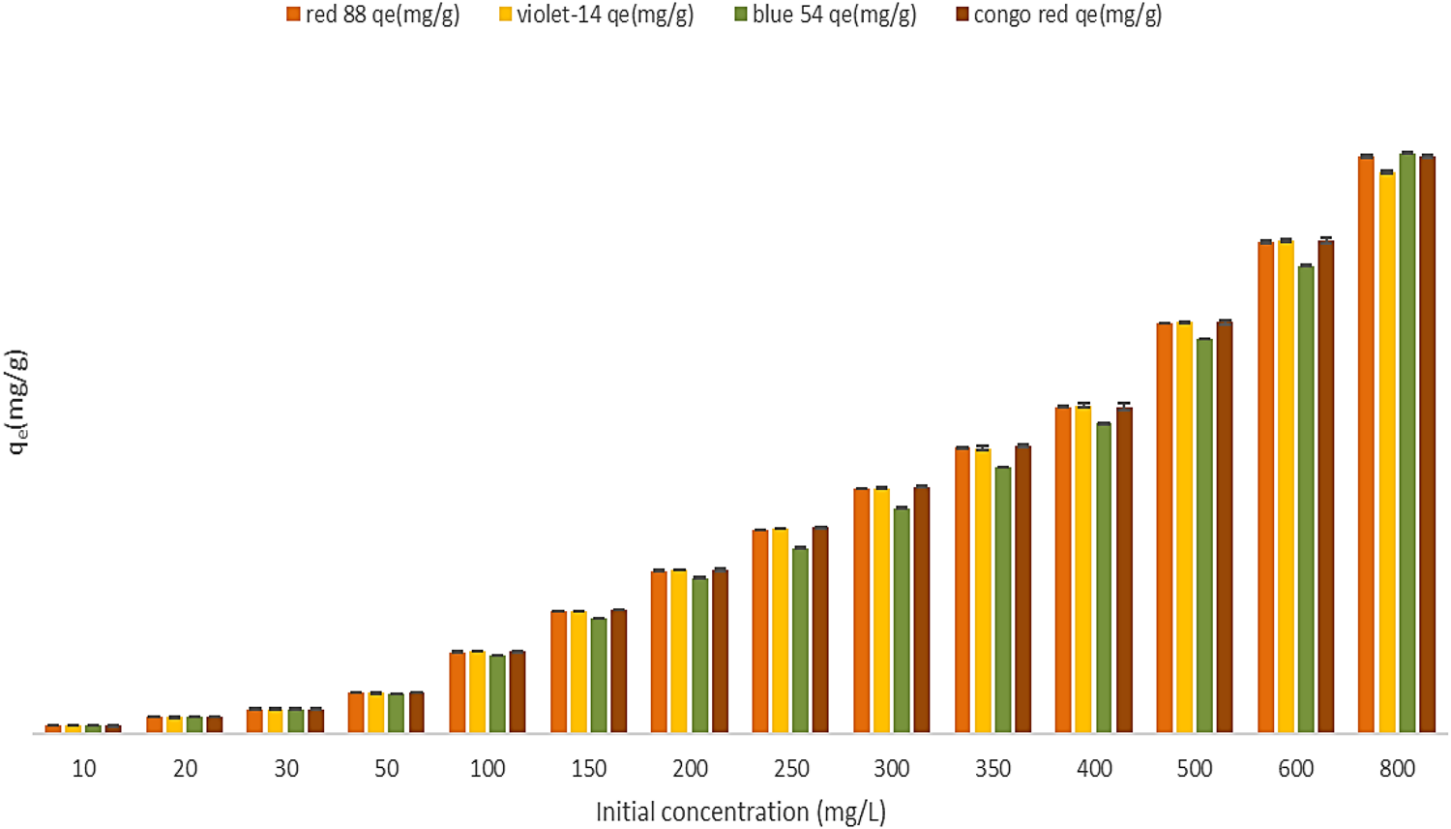
Effect of initial concentration (10–800 mg/L) on dye adsorption (Adsorbent mass 0.08 g/L, Contact time 20 min, Tem. 25–65 °C). The raw data for this figure is provided in the Supplementary file 1.

The initial dye concentrations play a critical role in overcoming dye mass transfer resistance between the aqueous and solid phases^36^. Therefore, increasing initial dye concentrations would increase dye's adsorption capacity. On the other hand, a dye molecule can travel from bulk liquids to the outside surface of adsorbent, indicating that the adsorption of dye onto adsorbent is dependent on the concentration of dye molecules at the time of adsorption. Therefore, at low initial concentrations the percentage removal of dye is greater, but at high initial concentrations it is smaller. In this study, the effects of dye concentration (from 10 to 800 mg/L) on adsorption were investigated at 25–65 °C and 0.08 g/L adsorbent concentration for 20 min. According to Fig. 10, incrementing the concentration of the initial dye from 10 to 800 mg/L, the ZPBIF-1 equilibrium sorption capacities increment from 12.5 to 875, 850, 877.68 and 875 mg/g for Acid red 88, Basic violet 14, Basic blue 54, and Congo red, respectively. Also, the removal efficiency reduced gradually from 100% to 88.75, 85.00, 87.76, and 88.75% with the increasing initial concentration of dyes Acid red 88, Basic Violet 14, Basic Blue 54, and Congo red, respectively.

### Adsorption isotherms

During the adsorption procedure, equilibrium isotherms were used to determine the relationship between the dyes adsorbed (Q_e_, mg/g) on ZPBIF-1 and the equilibrium concentration of the solution (C_e_, mg/L). The optimal conditions for dye adsorption were an adsorbent dosage of 0.08 g/L, pH = 2, and an equilibrium time of 20 min. Generally, adsorption isotherms present valuable information about surface properties, the tendency of adsorbents, and the adsorption mechanism^37^. In this study, Langmuir and Freundlich, Temkin, and Dubinin–Radushkevi isotherms were employed to describe the equilibrium of dyes adsorbed by ZPBIF-1. Among them, the Langmuir adsorption isotherm was the most suitable model. According to this model, adsorption occurs within homogeneous sites over the absorbent without any adsorbent transmission through the surface. The Langmuir isotherm equilibrium looks as below^38^:

3 $$\frac{1}{{q_{e} }} = \frac{1}{{Q_{\max } }} + \frac{1}{{bQ_{\max } }}\left( {\frac{1}{{C_{e} }}} \right)$$

**Figure 11:**
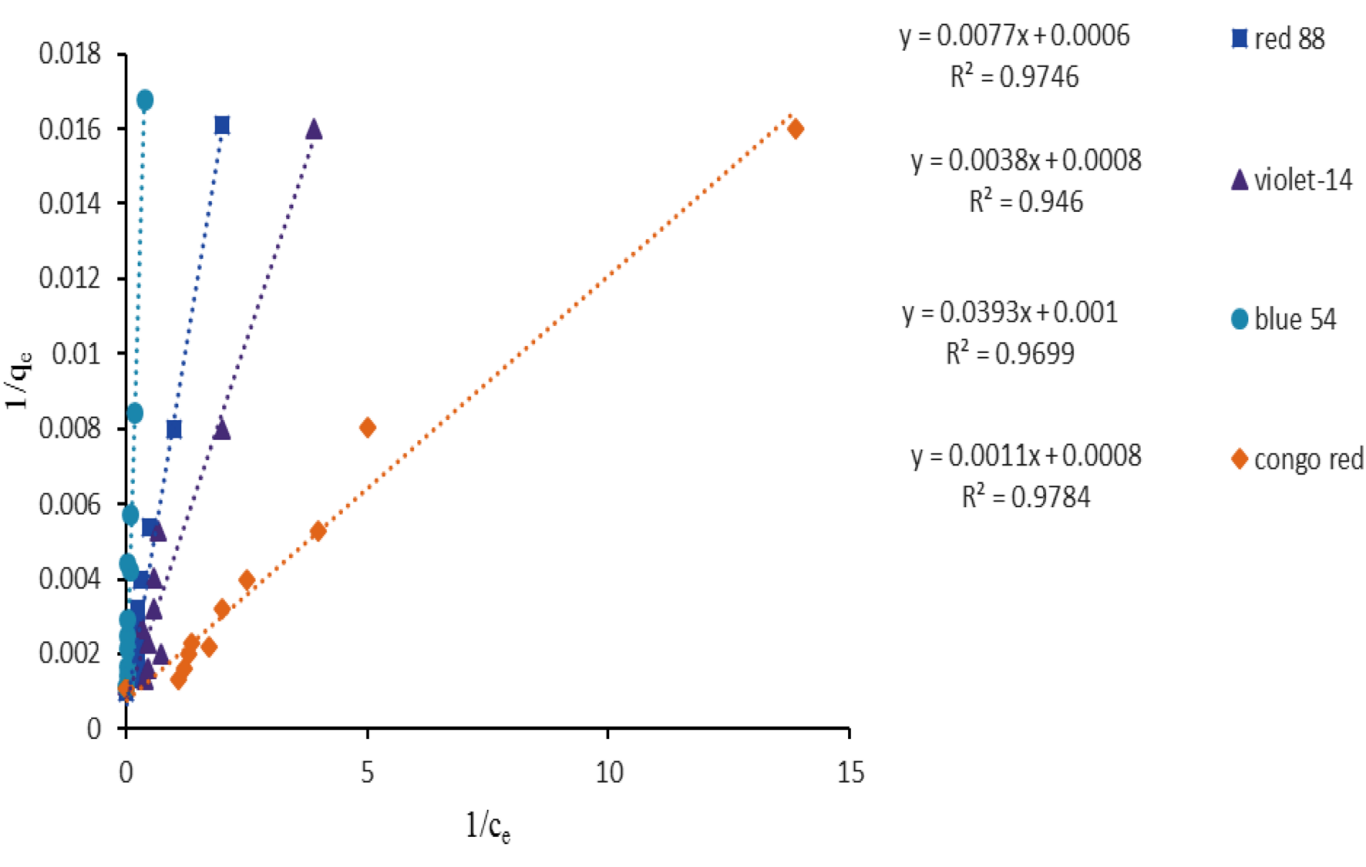
Linearized Langmuir isotherm for the adsorption of dyes by ZPBIF-1 composites. The raw data for this figure is provided in the Supplementary file 1.

In which, C_e_ (mg/L) and q_e_ (mg/g) represent the dye concentration in solution and the quantity of dye adsorbed per unit mass of ZPBIF-1 at equilibrium, respectively. Q_max_ (mg/g) represents the maximum adsorption capacity, as well as b (K_L_) (L/mg) describes the Langmuir constant for the adsorbent and adsorbate requirements. In the Langmuir model, the isotherm constants (K_L_, Q_max_, and R^2^) were found through linear plotting of 1/q_e_ against 1/C_e_ (Fig. 11).

It is indicated that the Langmuir model can acceptably provide the adsorption isotherm data. The Langmuir isotherm’s fundamental features are communicated based on equilibrium parameter (R_L_) or dimensionless constant separation factor as:

4 $${\mathbf{R}}_{\mathbf{L}}=1/(1+{\mathbf{K}}_{\mathbf{L}}{\mathbf{C}}_{0})$$

In which C_0_ (mg/L) is the most effective initial concentration of the adsorbent. The worth of R_L_ demonstrates the kind of the isotherm as linear (R_L_ = 1), unfavorable (R_L_ > 1), irreversible (R_L_ = 0), or favorable (0 < R_L_ < 1). Based on Table 1, all the R_L_ values were between 0 and 1, representing the favorable adsorption of dyes by ZPBIF-1. The utmost monolayer adsorption capacity Q_max_ was 1666.66, 1250, 1000, and 1250 mg /g for dyes, respectively.

**Table 1:** Linearized isotherm coefficients for dye adsorption (Initial dye concentration 50–800 mg/L, Adsorbent mass 0.08 g/L, Contact time 20 min, Tem. 25–65 °C).

Isotherm models	Isotherm parameters	Acid red 88	Basic Violet 14	Basic Blue 54	Congo red
Langmuir model	Q_m_ (mg/g)	1666.66	1250	1000	1250
	K_L_ (L/mg)	0.077	0.210	0.025	0.727
	R^2^	0.9746	0.946	0.9699	0.9784
Freundlich model	K_f_ (mg/g)	169.31	240.32	34.34	364.16
	n	2.02	2.59	1.32	3.00
	R^2^	0.6470	0.5473	0.9009	0.5585
Tempkin model	K_T_ (L/mg)	3.22	10.56	3.13	46.17
	b_T_ (J/mol)	17.10	18.92	11.00	20.72
	R^2^	0.6716	0.6158	0.7809	0.6992
	B_1_	164.27	130.92	225.21	119.56
Dubinin-Radushkevich model	Q_s_ (mg/ g)	471.11	462.66	407.15	617.20
	K_ad_ (mol^2^/J^2^)	0.0000007	0.00000009	0.000003	0.00000006
	E (kJ/mol)	0.845	2.357	0.408	2.886
	R^2^	0.6784	0.6228	0.6467	0.8624
Separation factor (R_L_)	C_0_ (mg/L) 50–800	0.206–0.015	0.086–0.005	0.444–0.047	0.026–0.001

The raw data for this table is provided in the Supplementary file 2.

Freundlich isotherm relies on reversible, monolayer, and non-ideal adsorption, which might be accustomed to multiple-layer adsorption on heterogeneous surfaces with disparate affinities and adsorption energy. This model could even be represented as:

5 $${q}_{e}={K}_{f}{{C}_{e}}^\frac{1}{n}$$

1/n and K_F_ are Freundlich constants associated with the adsorption intensity and adsorption capacity per unit concentration, respectively. The n and K_F_ are determined from the slope and axis intercept of the plot (Table 1). The value of n denotes the adsorption favorability. The character of adsorption is favorable when n ˃ 1. Equation (6) can be reset in linear form as:

6 $${\mathrm{logq}}_{\mathrm{e}}={\mathrm{logK}}_{\mathrm{f}}+\frac{1}{\mathrm{n}}{\mathrm{logC}}_{\mathrm{e}}$$

The linear plot of log q_e_ against log C_e_ was used to investigate the Freundlich isotherm of dye removal. The value of n in all cases was more significant than 1, indicating calm adsorption. The Temkin isotherm model assumes that the adsorption heat linearly diminished with expanding the coverage of the absorbent surface. One of the properties of this model is a uniform distribution of the binding energy. This isotherm can be written in linear form as^37^:

7 $${\mathrm{q}}_{\mathrm{e}}=\frac{\mathrm{RT}}{{\mathrm{b}}_{\mathrm{T}}}{\mathrm{lnk}}_{\mathrm{T}}+\frac{\mathrm{RT}}{{\mathrm{b}}_{\mathrm{T}}}{\mathrm{lnC}}_{\mathrm{e}}$$

in which b_T_ is that the Temkin constant represents the sorption point, (kJ/mol), and K_T_ is the equilibrium binding constant associated with the foremost energy (L/g). T is the temperature (K), and R is the gas constant (8.314 J/mol K). The Temkin isotherm compatibly with dye adsorption data through ZPBIF-1 was investigated by the linear plot of q_e_ against C_e_. Table 1 shows the isotherm constants (K_T_, R^2^, and B_T_). These values are but the Langmuir, and Freundlich values reveal that the Temkin isotherm features worse experimental data compared to the other isotherms.

The Dubinin–Radushkevich (DRK) isotherm is used to formulate the adsorption mechanism over a heterogeneous surface with a Gaussian energy distribution^39^. The empirical form is given by the Eq. (8). The DRK isotherm constant ε is obtained using Eq. (9). The model is mainly used to differentiate between physical and chemical adsorption of metal ions with its mean free energy E per adsorbent molecule as:

8 $${\mathrm{q}}_{\mathrm{e}}=({\mathrm{q}}_{\mathrm{s}})\mathrm{exp}(-{\mathrm{K}}_{\mathrm{ad}}{\upvarepsilon }^{2})$$

9 $$\begin{aligned} & \ln {\text{q}}_{{\text{e}}} = \ln ({\text{q}}_{{\text{s}}} ) - \left( {{\text{K}}_{{{\text{ad}}}} \varepsilon^{2} } \right) \\ & \varepsilon = {\text{RT}}\ln \left[ {1 + \frac{1}{{{\text{C}}_{{\text{e}}} }}} \right] \\ \end{aligned}$$

10 $$\mathrm{E}=\left[\frac{1}{\sqrt{2{\mathrm{B}}_{\mathrm{DR}}}}\right]$$

here, q_e_ is the amount of adsorbate (mg/g); q_s_ shows the theoretical saturation capacity (mg/g); K_ad_ and B_DR_ are the DRK isotherm constants (mol^2^/kJ^2^), and ε is the DRK isotherm constant. The gas constant (8.314 kcal/mol K), adsorbate equilibrium concentration (mg/L), and absolute temperature (K) are represented by R, Ce, and T, respectively.

The parameters of the models' isotherms are shown in Table 1. Based on the correlation coefficients (R^2^) provided by the isotherm models, the Langmuir isotherm possesses the maximum correlation indicating dye adsorption on a homogeneous surface.

In Table 2, the adsorption capacity of ZPBIF-1 was 1666.66, 1250, 1000, and 1250 mg/g, substantially more signicant than the values reported for most other adsorbents. The superior adsorption properties of ZPBIF-1 are attributed to its unique structure, as described below.

1. ZPBIF-1 nanoparticles are distributed uniformly. It not only has adsorption sites by Zn, but also provides organic framework sites for adsorption.
2. It was found that ZPBIF-1 has a much higher surface area than simply agglomerated ZIF-8 nanoparticles
3. Due to the aromatic rings of the ligands of 2-Phenyl benzimidazole, ZPBIF-1 creates an π–π interaction with dyes.

**Table 2:** Comparison of dye adsorption capacities for different adsorbents.

Adsorbents samples	q_max_ (mg/g)	Refs.
**Acid red 88**		
Bituminous coal	26.1	^40^
Activated carbon	109.0	^40^
Anion exchange membrane	42.01	^41^
Raw alunite	20.98	^42^
MNZnFe	111.1	^43^
ZPBIF-1	1666	This study
**Basic Violet14**		
Deoiled soya	12.03	^44^
Bottom ash	6.39	^44^
Curcuma angustifolia Scales	208.33	^45^
Calophyllum inophyllum Shells	1416.43	^46^
Theobroma cacao Shells	980.39	^46^
ZPBIF-1	1250	This study
**Congo red**		
ZIF-67	714.3	^47^
Chitosan hydrogel beads impregnated with cetyl trimethyl ammonium bromide	352	^48^
Microwave-assisted ZIF-8	167	^49^
Activated carbon coffee waste (ACCW)	90.9	^50^
Activated carbon from java citronella distillation waste	3.03	^51^
ZPBIF-1	1250	This study
**Basic Blue 54**		
Sludge biomass	86.6	^52^
ZPBIF-1	1000	This study

The higher adsorption capacity of this adsorbent is related additionally to the porous structure, their great specific extent, and more active adsorption sites. It’s concluded that the ZPBIF-1 possesses a relatively higher adsorption capacity representing its feasibility, and applicability as a low-cost, convenient, and effective adsorbent in the adsorption method to urge obviate dyes.

### Adsorption kinetics study

The prediction of sorption rate is a key factor in designing batch adsorption procedures, and it is affected by adsorption kinetics. Chemical kinetic models are orientated by the adsorbent's chemical and physical properties, and their assessment is critical to providing insightful information about the factors influencing the reaction speed^53^. The experimental conditions were conducted at the enhanced points as solution pH = 2, the adsorbent dosage of 0.08 g/L, and initial dye concentrations of 50 and 250 mg/L. Furthermore, six adsorption models were introduced in this study.

- First-order reaction model in terms of the solution concentration
- Lagergren pseudo-first-order equation in terms of the solid capacity^54^
- Second-order reaction model oriented by the solution concentration
- Pseudo-second-order reaction model of Mckay and H_o_, in terms of the solid phase sorption
- Intra-particle diffusion^55^
- Using Elovich models, the kinetics of dye adsorption over the prepared adsorbents were evaluated

The integrated form of the first-order rate equation in terms of the solution concentration can be stated as:

11 $${\mathrm{lnC}}_{\mathrm{t}}={\mathrm{lnC}}_{0}-{\mathrm{k}}_{1}\mathrm{t}$$

In Eq. (11), the plot of ln Ct versus time (t) shows the linear plot of the first-order equation.

Using the best-fit line, the coefficient value (R^2^) was determined (Table 3). The pseudo-first-order kinetic model can be integrated in terms of the solid capacity for sorption analysis as:

**Table 3:** Kinetics constants for the adsorption of dyes by ZPBIF-1 (Contact time = 20 min, Tem = 25–65 °C, Adsorbent mass = 0. 08 g/L, Initial concertation 50 and 250 mg/L, Stirring = 400 rpm and pH = 2).

	Acid Red 88		Basic Violet 14		Basic blue 54		Congo red	
**Pseudo-first-order model**								
C_0_ (mg/L)	50	250	50	250	50	250	50	250
q_e,exp_(mg/g)	61.87	307.55	62.18	310.30	59.37	281.25	62.41	311.87
q_e,cal_ (mg/g)	55.63	370.59	53.57	183.71	61.90	252.29	59.24	270.20
K_1_/(min)	0.1376	0.3894	0.1704	0.6632	0.3614	0.2998	0.4054	0.5918
R^2^	0.9333	0.9732	0.9160	0.9304	0.9983	0.9845	0.9981	0.9930
**First-order model**								
C_0_	64.80	185.04	61.75	107.66	34.80	183.60	36.88	102.53
K_1_	0.2066	0.2208	0.2384	0.2548	0.1554	0.1185	0.3401	0.3329
R^2^	0.8630	0.9301	0.888	0.7745	0.8722	0.8470	0.9774	0.8386
**Pseudo-second-order model**								
q_e,cal_ (mg/g)	59.88	312.5	60.97	312.5	58.82	277.77	62.50	312.5
K_2_ (g/min mg)	0.0228	0.0256	0.0389	0	0.0741	0.0648	0.3657	1.024
R^2^	0.9798	0.9994	0.9926	1	0.9979	0.9999	1	1
**Second-order model**								
C_0_	2.94	89.28	1.44	10.39	28.01	142.85	0.42	28.32
K_2_	0.0806	0.0138	0.1575	0.029	0.0199	0.0018	0.7185	0.1314
R^2^	0.5178	0.8610	0.5106	0.7872	0.9682	0.9310	0.7514	0.9737
**Intra-particle diffusion model**								
K_p_ (g/min.mg)	7.6325	10.3320	4.6708	0.0381	1.4859	6.9672	0.6844	0.3658
C (mg/g)	26.29	263.53	40.30	310.13	52.95	250.35	59.45	310.42
R^2^	0.8209	0.7934	0.7896	0.9977	0.8812	0.9930	0.8846	0.8637
**Elovich constants**								
R^2^	0.7865	0.8273	0.7554	0.9916	0.9063	0.9984	0.9094	0.892
α (mg/g min)	50.6321840032	5,454,780.13 12,788	508.2847556925	∞	169,642,386.81368	1,169,223,763.5818	3.06752219E + 190	1.36661039E + 191
ß (g/mg)	0.070	0.050	0.115	13.90	0.350	0.075	0.761	1.421

The raw data for this table is provided in the Supplementary file 2.

12 $$\mathrm{ln}\left({\mathrm{q}}_{\mathrm{e}}-{\mathrm{q}}_{\mathrm{t}}\right)={\mathrm{lnq}}_{\mathrm{e}}-{\mathrm{ks}}_{1}\mathrm{t}$$

Equation (12) represents a linear plot of ln (q_e_ − q_t_) against time (t), denoting a pseudo-first-order. qt (mg/g) shows the absorption quantity at time t, while ks_1_ denotes the constant of the equilibrium rate pseudo-first-order kinetic (/min). In the plot of ln (q_e_ − q_t_) against t, the straight line represents this kinetic model sufficiency presenting an association between the intercept and slope. The model parameters (ks_1_ and estimated q_e_) can be determined, respectively. The kinetic constants (ks_1_, R^2^, and (q_e_)_Cal_.) are represented in Table 3.

Second-order reaction model.

The integrated second-order rate equation is extensively utilized for the adsorption process, which can be represented mathematically as Eq. (13):

13 $$\frac{1}{{\mathrm{C}}_{\mathrm{t}}}-\frac{1}{{\mathrm{C}}_{0}}={\mathrm{k}}_{2}\mathrm{t}$$

It shows that 1/C_t_ against time (t) is a linear plot, which indicates that the reaction rate is second order.

**Figure 12:**
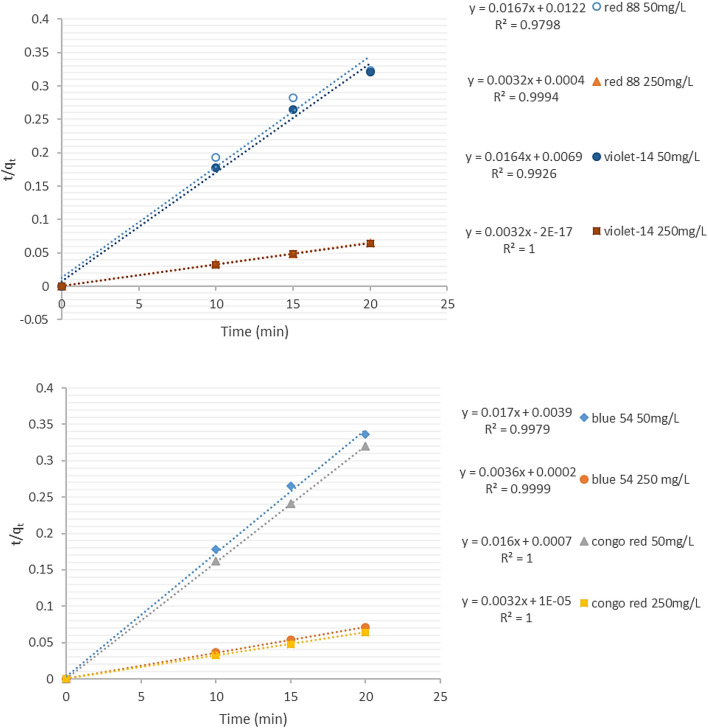
Kinetics plot for the adsorption of dyes by ZPBIF-1. The pseudo- second-order model (Contact time = 20 min, Tem = 25–65 °C, Adsorbent mass = 0. 08 g/L, Initial concertation 50 and 250 mg/L, Stirring = 400 rpm and pH = 2).

The pseudo-second-order model adsorption kinetic depends upon the solid adsorption capacity. Moreover, contrary to other models, it can estimate the absorption performance throughout the absorption range. The linear form of the model is:

14 $$\frac{\mathrm{t}}{{\mathrm{q}}_{\mathrm{t}}}=\frac{1}{{\mathrm{k}}_{2}{\mathrm{q}}_{\mathrm{eq}}^{2}}+\frac{\mathrm{t}}{{\mathrm{q}}_{\mathrm{eq}}}$$

which, q_eq_ and q_t_ represent the numbers of dye adsorbed (mg/g) at equilibrium and time t, respectively; K_2_ shows the pseudo-second-order kinetic model’s equilibrium rate constant (g/mg/min). Linear plotting t/q_t_ against t was used to investigate the compatibility of pseudo-second-order kinetics on dye removal at various adsorbent dosages from single systems. The value of q_eq_ is obtained from the slope of the t/q_t_ against t in the plot (Fig. 12), and K_2_ is determined from the initial sorption rate value. Results showed that the adsorption process conformed better to the pseudo-second-order model. There was a linear plot with the highest correlation coefficients in this model (Table 3). Considering the coefficient of the determinant of the value, the pseudo-second-order reaction kinetic model was followed by the adsorption processes well compared to the other three kinetic models.

Adsorption is often a multi-stage process, including transferring aqueous solute molecules to the solid particles' surface, where they penetrate the pores (slower step). The diffusion mechanism is not determined by first and second-order kinetic models. Hence, it is preferred to use the intraparticle diffusion model to evaluate the results of kinetics as follows^56^:

15 $${\mathrm{q}}_{\mathrm{t}}={\mathrm{k}}_{\mathrm{p}}{\mathrm{t}}^{1/2}+\mathrm{I}$$

where Kp (mg/g min^1/2^) denotes the intraparticle diffusion rate constant, and I is the intercept. The intraparticle diffusion kinetics constants (R^2^, I, and K_p_) attained by linear plotting of q_t_ against t^1/2^ are presented in Table 3.

Another kinetic model equation in terms of the adsorption capacity is the Elovich equation by Eq. (16)^57^:

16 $${\mathrm{q}}_{\mathrm{t}}=\frac{1}{\upbeta }\mathrm{ln}(\mathrm{\alpha \beta })+\mathrm{ln}(\mathrm{t})$$

α and β respectively represent the Elovich constants denoting the initial adsorption rate (g/mg min) and the desorption constant (mg/g min). The Elovich constants are attained from the graphs of q_t_ versus ln t (Table 3).

According to the results from Table 3, the Pseudo-second order model is the best fitting model representing the higher correlation coefficient Acid red88, Basic violet14, Basic blue54, and Congo red, R^2^ = 50 mg/L (0.9798, 0.9926, 0.9979, 1.0), and 250 mg/L (0.9994, 1.0, 0.9999, 1.0) for the adsorption of dyes by ZPBIF-1.

Thermodynamics study and effects of temperature.

Due to the importance of temperature in the adsorption process, its impacts on the adsorption of dyes on ZPBIF-1 were examined at 24, 45, and 65 °C, and pH = 2 and 100 mg/L of initial dye concentration. The thermodynamic parameters such as entropy (ΔS°), Gibbs free energy (ΔG°), and enthalpy (ΔH°) were determined as:

16 $${\mathrm{LnK}}_{\mathrm{d}}=\Delta {\mathrm{S}}^{\circ }/\mathrm{R}-\Delta {\mathrm{H}}^{\circ }/\mathrm{RT}$$

17 $${\mathrm{K}}_{\mathrm{d}}=({\mathrm{C}}_{\mathrm{o}}-{\mathrm{C}}_{\mathrm{a}})*\mathrm{V}/({\mathrm{C}}_{\mathrm{o}}*\mathrm{m})$$

18 $$\Delta {\mathrm{G}}^{0}=-{\mathrm{RTLnK}}_{\mathrm{d}}$$

where T represents the temperature in Kelvin, and R is the gas constant (8.314 J/mol K). K_d_ shows the distribution coefficient determined by Eq. (17). C_0_ denotes the initial dye concentration in solution (mg/l), m represents the adsorbent mass (g), V is the solution volume, C_e_ is the equilibrium concentration (mg/l), and Q_e_ is the quantity adsorbed per unit adsorbent mass at equilibrium (mg/g). Entropy (ΔS°) and enthalpy (ΔH°) were computed from the intercept and slope of the Van’t Hoff plot of ln q_e_/C_e_ versus 1/T. To calculate the value of Gibbs free energy (ΔG°), Eq. (18) was used (Table 4).

**Table 4:** Thermodynamic parameters for the adsorption of dyes on ZPBIF-1.

ΔS (J/mol K)	ΔH (Kj/mol)	ΔG (Kj/mol)	(k)	(Acid red88) (PPM)
124.37	29.09	− 8.44	298	100/mg/L
		− 10.86	318	
		− 13.51	338	

ΔS (J/mol.K)	ΔH (Kj/mol)	ΔG (Kj/mol)	(k)	(Basic Violet 14) (PPM)
− 71.20	− 35.04	− 13.65	298	100 mg/L
		− 10.99	318	
		− 10.75	338	

ΔS (J/mol.K)	ΔH (Kj/mol)	ΔG (Kj/mol)	(k)	(Basic Blue54) (PPM)
− 5.63	− 9.74	− 7.82	298	100 mg/L
		− 7.98	318	
		− 7.55	338	

ΔS (J/mol.K)	ΔH (Kj/mol)	ΔG (Kj/mol)	(k)	(Congo red) (PPM)
− 254.04	− 91.34	− 15.92	298	100 mg/L
		− 5.42	318	
		− 5.71	338	

The raw data for this table is provided in the Supplementary file 2.

The negative value of ΔH° represents the exothermic adsorption of Basic Violet14, Basic Blue 54, and Congo red onto ZPBIF-1. Moreover, the negative value of ΔS° suggests the reduction of randomness at the solid-solution interface during the adsorption procedure^55^. During the procedure, the mobility of dye molecules was increased by increasing the temperature, which caused them to escape from the solid to the liquid phase. Acid red 88 exhibited endothermic properties during its adsorption process, as indicated by its positive value of ΔH°. However, randomness increased at the solid-solution interface, as shown by its positive value of ΔS°. The negative values of Gibb’s free energy ΔG° represent the spontaneous adsorption process for all dyes.

### Adsorption mechanism

**Figure 13:**
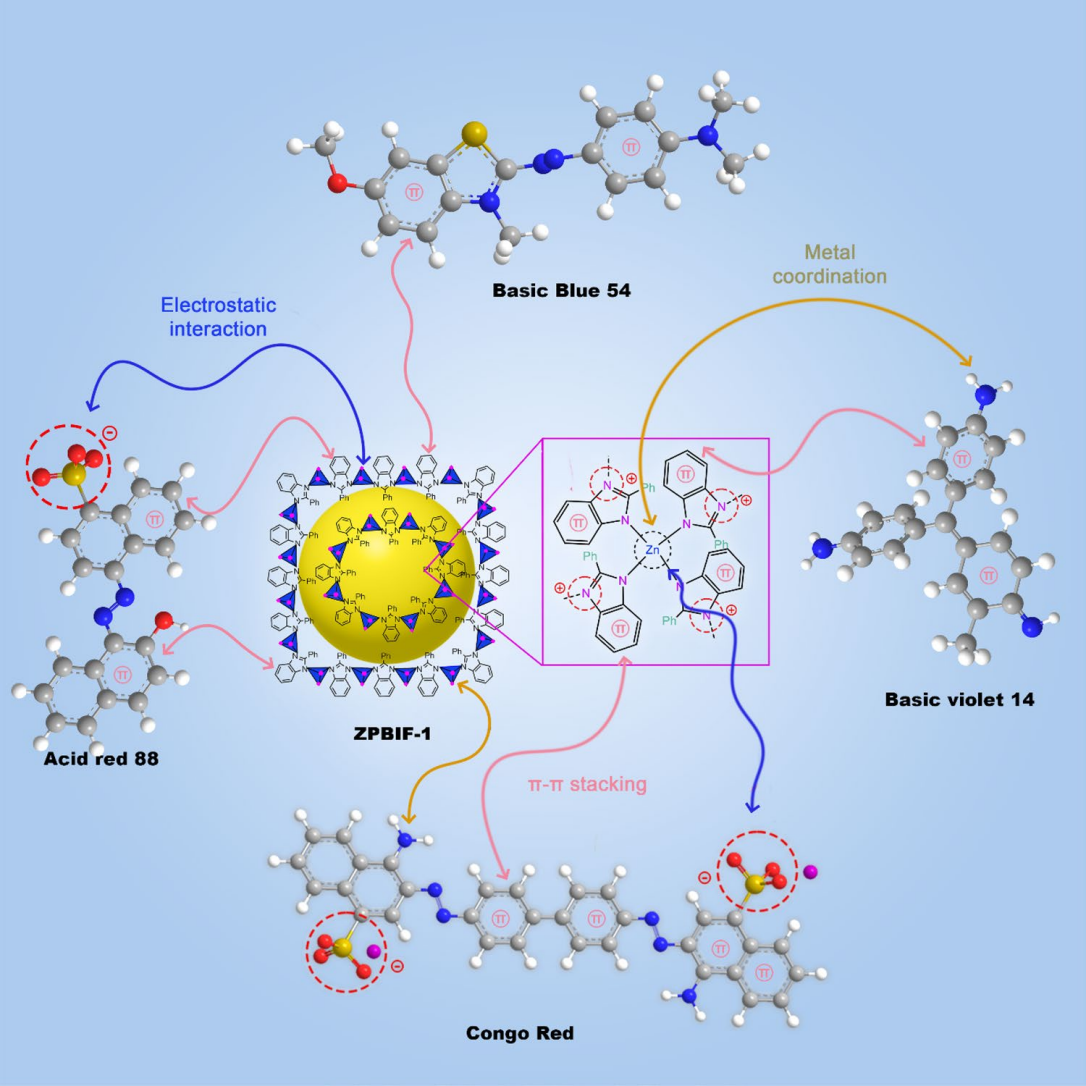
Plausible adsorption mechanism of ZPBIF-1 towards organic dyes.

Different interactions are responsible for the adsorption mechanism of organic dyes on the ZPBIF-1 surface. The electrostatic attractions between the positively charged Zn groups occurred on the ZPBIF-1 surface with negatively charged sulfonate (SO_3_^-^) groups of Acid red 88 and Congo red dyes. Molecules were readily adsorbed on the positively charged ZPBIF-1, and the open sites within the ZPBIF-1 were simply complexed with NH_2_. This was probably due to a soft–soft interaction of the metal ions of ZPBIF-1 with the amino groups of the dyes^58^. Furthermore, there may be an π–π attraction between the dye molecules and phenyl benzimidazole ligands^59^. In total, three main types of interactions were considered in the adsorption mechanism, including π–π attraction, electrostatic interactions, and metal coordination. The proposed adsorption mechanism was presented as a schematic diagram (Fig. 13).

To demonstrate the interactions between dyes and ZPBIF-1, the FTIR spectra of adsorbent before and after adsorption of Congo red were compared. For example, the peak at 1388.78/cm in ZPBIF-1 was indicative of C–N vibration, which changed enormously after adsorption and became a dual peak in 1400–1460/cm. The peaks at 697.63/cm and 750.70/cm in ZPBIF-1 represented Z–N and C=C vibrations were reduced to 650.50/cm and 744.20/cm after adsorption of Congo red. Moreover, the peaks at 1061.54, 1178.78, and 1224.99/cm for S=O in Congo red were changed after adsorption on the ZPBIF-1 surface.

**Figure 14:**
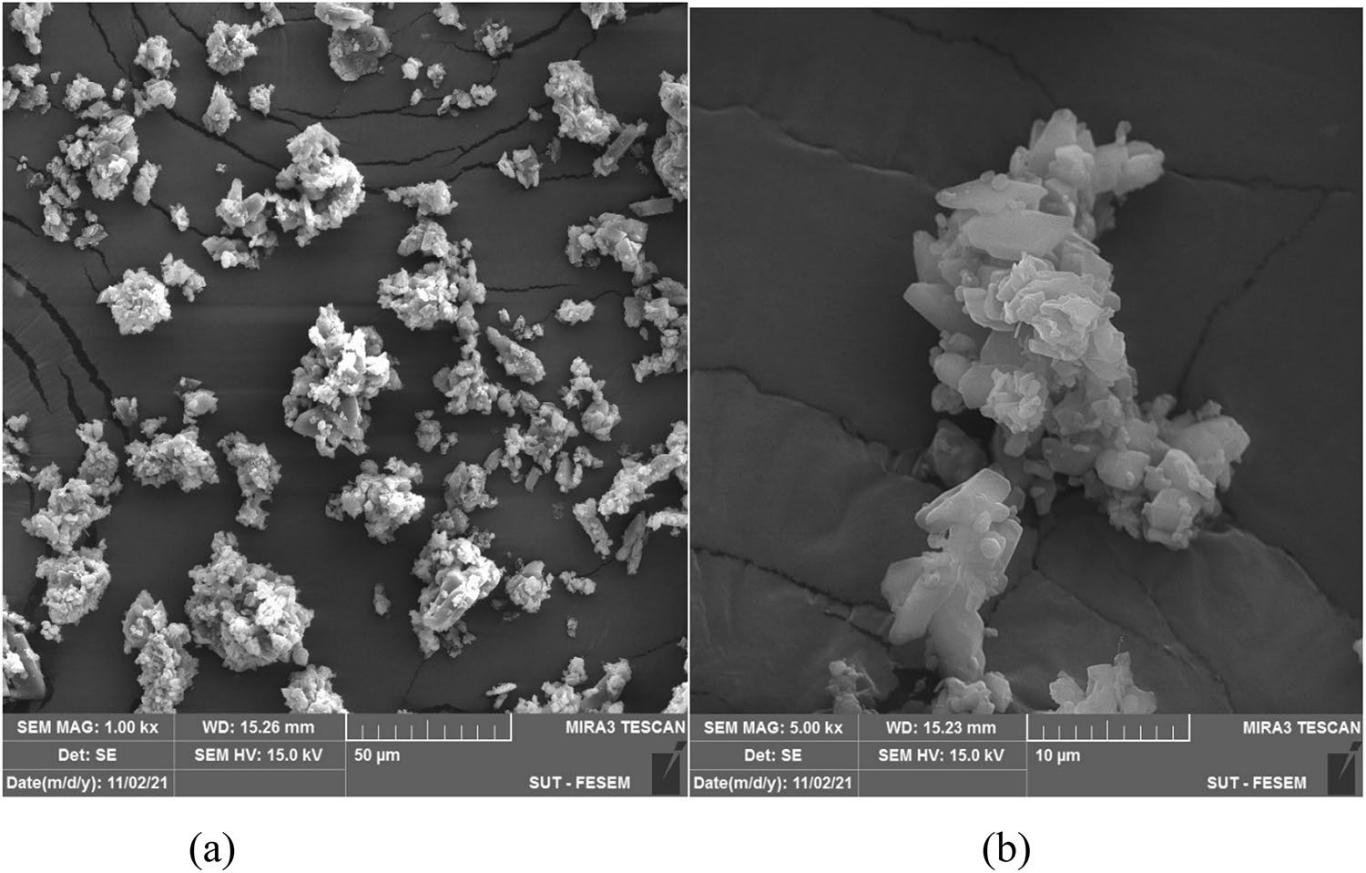
SEM images of ZPBIF-1 after adsorption of Acid red 88 (**a**, **b**).

SEM images of ZPBIF-1 after adsorption of Acid red 88 are shown in Fig. 14, which can compare to the unused ZPBIF-1 (Fig. 5). The morphology of the unused ZPBIF-1 showed regular rods with hexagonal faces. However, its surface after adsorption of Acid red 88 dye was morphologically irregular, which is not surprising since the dye molecules adsorbed on the ZPBIF-1 surface caused morphological changes. These results showed that ZPBIF-1 could adsorb Acid red 88 molecules well.

XRD patterns of ZPBIF-1 composites before and after dye adsorption showed similar peak shapes, revealing the failure of adsorption to alter the crystalline form of ZPBIF-1.

### Regeneration and recycling of ZPBIF-1 nanocomposites

**Figure 15:**
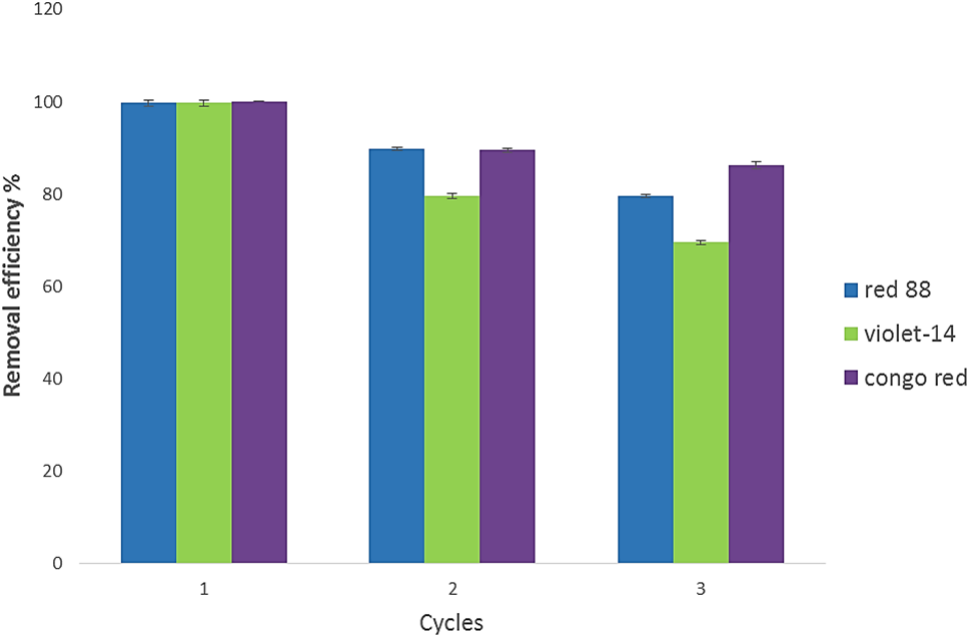
Recyclability study of ZPBIF-1 towards dyes. The raw data for Fig. 14 is provided in the Supplementary file 1.

In large-scale functional applications, recyclability and stability of adsorbents play an important role in reducing the material cost of water treatment. The practical potential for adsorbents can be evaluated substantially based on their recyclability. Here, the ZPBIF-1 adsorbent was recovered and used in 3 cycles for dye removal. The results of the reusability of ZPBIF-1 are demonstrated in Fig. 15. It should be noted that even after 3 cycles, the percent removal was above 80%.

### Removal of dyes from wastewater sample

The applicability of the developed adsorbent was evaluated using a textile wastewater sample. The wastewater sample was filtered using a 0.45-mm pore size membrane filter to remove suspended particulate matter. One solution containing 50 mg/L of acid red 88 in wastewater was prepared as an actual sample. Then, 0.016 g of ZPBIF-1 was added to 100 mL wastewater at pH = 2. The mixed solution was shacked for 20 min. Because of the matrix effect, the tests were performed through the standard addition method, and the initial and final concentrations of the target species (before and after removal with the recommended procedure) were determined. For this aim, four 25 mL volumetric flasks were each filled with 20 mL of wastewater samples. Then, the different amounts of the standard acid red 88 solutions (0, 0.25, 0.5, and 0.75 mL of 1000 mg/L) were added, and the solutions in the flasks were diluted to the mark and mixed well. The amounts of dye in the solution were determined by the UV–Vis spectrophotometry method, and the absorption versus standard concentration was plotted. A simple Linear Least Squares analysis was conducted using the slope and intercept functions of Microsoft Excel. To find the initial concentration of the unknown, the value of X at y = 0 from y = mX + b was calculated. As shown in Table 5, the proposed method could be applied successfully for the removal of acid red 88 from an actual sample of textile wastewater with acceptable efficiency. ZPBIF-1 had a removal efficiency of 92.9% for the real sample.

**Table 5:** Removal of Acid Red 88 dye from textile wastewater sample.

Dye	Initial concentration (mg/L)	Final concentration (mg/L)	% Removal
Acid Red 88	50	3.55	92.9

The raw data for this table is provided in the Supplementary file 2.

Biodegradability, as well as biological oxygen demand (COD and BOD), were examined on an actual wastewater sample. The COD level decreased from the initial concentration of 1910–63 mg/L. Similarly, the BOD level gradually reduced from the initial concentration of 810–27 mg/L. Based on the acceptable amounts for irrigation and agricultural uses, 200 and 100 mg/L, respectively, the performance of this adsorbent has been evaluated very well, and the treated effluent can be reused. As a result, during the adsorption process, the organic portion of the dye molecules may be destroyed, decomposed, and even adsorbed.

## Conclusion

A new MOF, Tetrakis (2-phenylbenzoimidazol-1-yl) zinc (ZPBIF-1), was successfully synthesized by the solvothermal method. According to the results of response surface methodology-based optimization, the optimal conditions to eliminate Acid red88, Basic violet14, Basic blue54, and Congo red were Co = 20 mg/L, contact time = 20 min , adsorbent dosage = 0.08 g/L, and pH = 2. In comparison with other adsorbents, ZPBIF-1 showed superior adsorption capacity at 1666.66, 1250, 1000, and 1250 mg/g for the above dyes. The adsorption kinetics followed the pseudo-second-order models for all organic dyes. In the meantime, according to the adsorption isotherms, all colors follow the Langmuir model. The exceptional adsorption properties of ZPBIF-1 can be attributed largely to the unique structure and interaction of active functional groups, such as electrostatic interaction, hydrogen bonding, π–π conjugation, and coordination effect of zinc. The thermodynamic parameters showed that adsorption on ZPBIF-1 was spontaneous and thermodynamically favorable. The dye adsorption for Basic violet 14, Basic blue 54, and Congo red by ZPBIF-1 was exothermic, while the dye adsorption for Acid red 88 was endothermic.

ZPBIF-1’s unique properties, such as its high recycling capacity, the number of aromatic rings, high removal efficiency, and maximum adsorption capacities (1666.66–1250 mg/g) make it an effective dye adsorbent that can be used for the removal of dye from wastewater.

## Supplementary Information

Supplementary Information 1.

Supplementary Information 2.

Supplementary Information 3.

Supplementary Information 4.

Supplementary Information 5.

Supplementary Information 6.

## Data Availability

All data generated or analyzed during this study are included in this published article and its supplementary information files (Supplementary files 1–6). Requests for material and additional data should be made to the corresponding authors.
