# Supplementary material for: Design, synthesis, and characterization of a novel Zn(II)-2-phenyl benzimidazole framework for the removal of organic dyes
Source: Sci Rep. 2022 Jul 20;12:12431. doi: 10.1038/s41598-022-16753-8 (PMC9300708; doi:10.1038/s41598-022-16753-8)
Supplement: Supplementary file 1 — Supplementary Information 1. [file 41598_2022_16753_MOESM1_ESM.docx]

**Novel Zn (II)-2-phenyl benzimidazole framework for the efficient removal of organic dyes: synthesis, characterization, adsorption, kinetic, and thermodynamic studies**

Shabnam Alibakhshi^1^, Ashraf S. Shahvelayati^a1^, Shabnam Sheshmani^1^, Maryam Ranjbar^2^, Saeid Souzangarzadeh^1^

*^1^Department of Chemistry, College of Basic Sciences, Yadegar-e- Imam Khomeini (RAH) Shahre Ray Branch, Islamic Azad University, Tehran, Iran.* * Corresponding Author: [avelayati@yahoo.com](mailto:avelayati@yahoo.com), [a_shahvelayati@iausr.ac.ir](mailto:a_shahvelayati@iausr.ac.ir)

*^2^Department of Chemical Technologies, Iranian Research Organization for Science and Technology (IROST), Tehran, Iran*

**Supplementary file 1: The raw Data for figures 2, 4, 7, 8, 9, 10, 11, 12, 15**


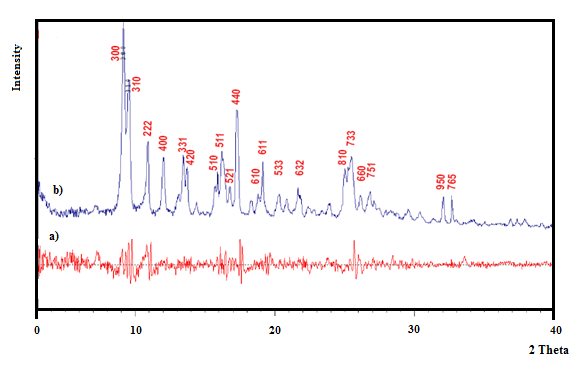


**Fig. 2.** a) The simulated PXRD pattern of ZIF-11, b) XRD pattern of Zn (II)-2-phenyl

benzimidazole framework (ZPBIF-1).


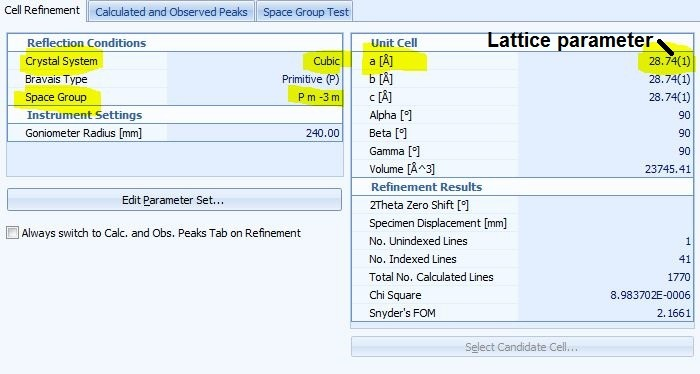


**Peak List**

Pos.[°2Th.] Height [cts] d-spacing [Å] Rel. Int. [%] FWHMLeft[°2Th.]

9.140(1) 6482(91) 9.66739 100.00 0.248(5)

9.514(2) 4705(86) 9.28834 72.59 0.208(5)

10.867(2) 2583(67) 8.13502 39.85 0.176(6)

11.997(2) 2109(57) 7.37101 32.54 0.194(7)

13.07(1) 553(58) 6.76661 8.53 0.24(3)

13.439(3) 1954(81) 6.58339 30.15 0.199(9)

13.697(3) 1672(105) 6.45989 25.80 0.135(9)

14.369(6) 449(41) 6.15920 6.92 0.12(1)

15.699(4) 1039(61) 5.64023 16.02 0.14(1)

15.881(2) 1757(134) 5.57617 27.10 0.043(4)

16.242(3) 2180(43) 5.45289 33.63 0.348(9)

16.760(3) 966(69) 5.28549 14.90 0.110(7)

17.272(1) 4257(62) 5.13004 65.68 0.181(3)

18.289(5) 547(42) 4.84693 8.43 0.16(1)

18.806(6) 682(41) 4.71496 10.52 0.21(2)

19.106(2) 1933(68) 4.64139 29.82 0.136(7)

20.243(5) 769(29) 4.38318 11.86 0.27(1)

20.823(4) 633(39) 4.26246 9.77 0.17(1)

21.626(9) 782(54) 4.10599 12.07 0.20(2)

21.81(1) 501(69) 4.07091 7.73 0.13(3)

22.39(1) 251(37) 3.96751 3.88 0.21(4)

22.75(3) 108(30) 3.90506 1.66 0.24(6)

23.862(5) 488(31) 3.72611 7.52 0.18(2)

24.993(3) 1499(49) 3.55997 23.13 0.241(8)

25.416(3) 2146(37) 3.50158 33.10 0.375(9)

26.113(4) 650(51) 3.40979 10.03 0.17(2)

26.755(4) 841(35) 3.32931 12.97 0.24(1)

27.098(5) 565(71) 3.28804 8.72 0.10(2)

27.40(1) 250(32) 3.25201 3.85 0.21(4)

28.29(1) 239(29) 3.15240 3.69 0.23(4)

28.71(1) 178(36) 3.10646 2.75 0.15(4)

29.518(8) 333(24) 3.02368 5.13 0.27(3)

30.373(8) 309(22) 2.94049 4.77 0.29(3)

31.33(1) 158(21) 2.85252 2.44 0.19(3)

32.017(2) 1064(45) 2.79318 16.42 0.15(1)

32.649(1) 1455(57) 2.74049 22.45 0.066(3)

34.09(2) 155(10) 2.62754 2.40 0.51(3)

35.00(1) 126(40) 2.56170 1.94 0.12(6)

36.842(7) 277(37) 2.43765 4.27 0.12(2)

37.32(1) 204(31) 2.40743 3.14 0.15(3)

37.871(8) 250(23) 2.37381 3.86 0.24(3)

39.074(9) 208(35) 2.30342 3.21 0.13(3)

Sum of net area: 12871

Sum of total area: 64783

**Crystallinity(%): 20**


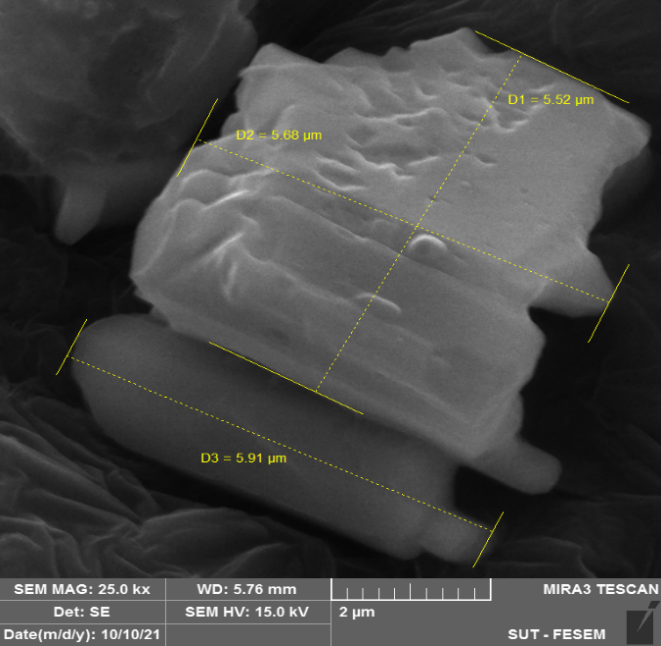

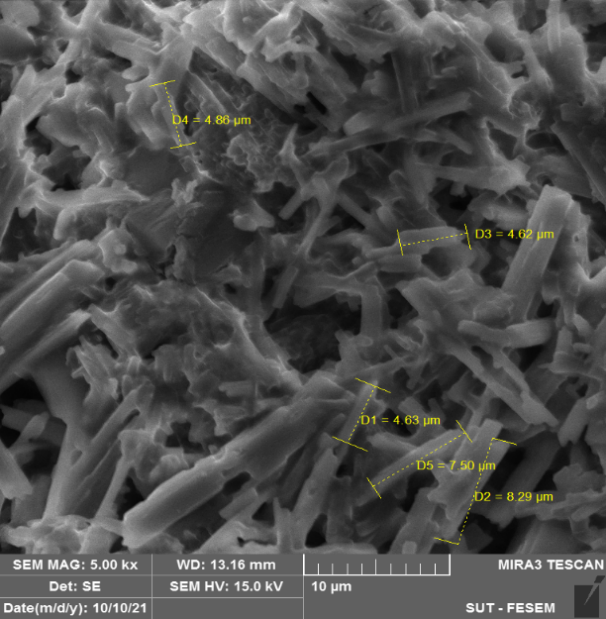

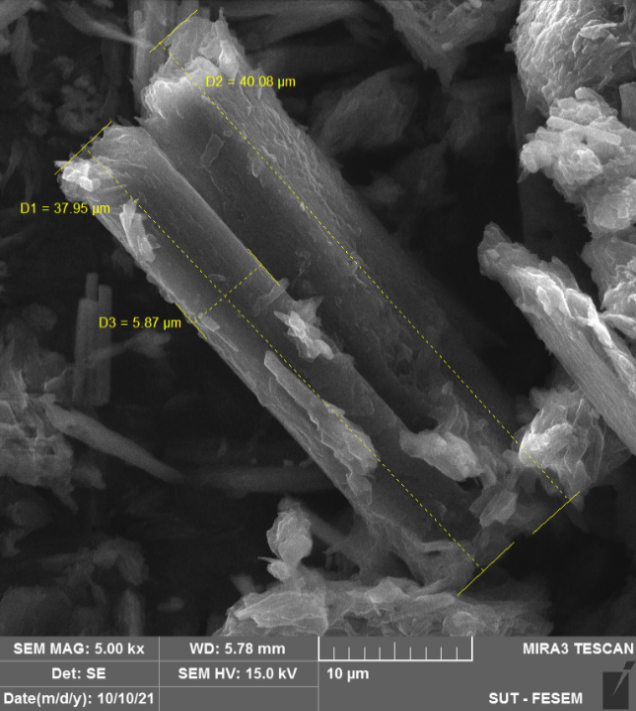

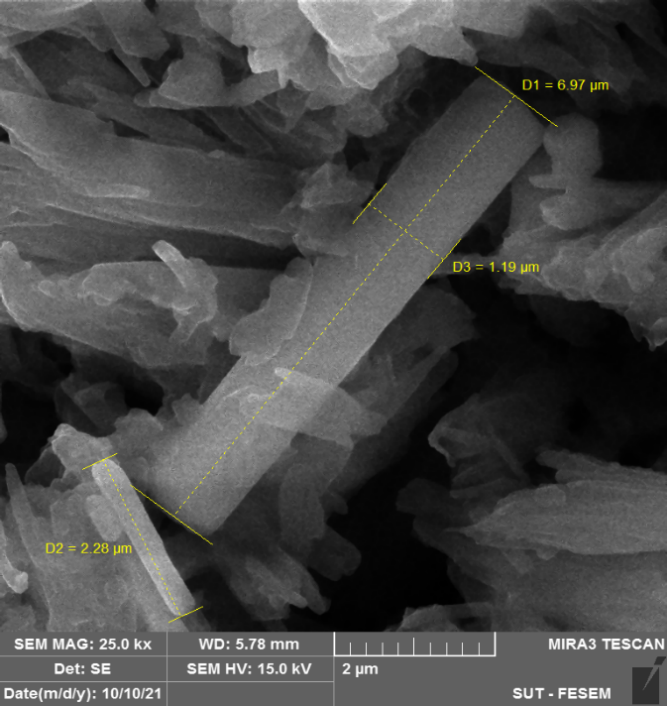


**Fig. 3.** Scanning electron microscopy images of ZPBIF-1


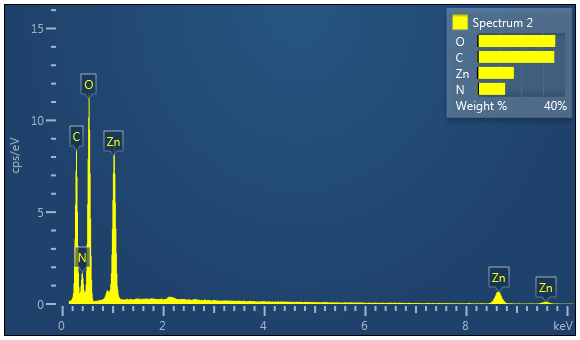


**Fig. 4.** Energy-dispersive X-ray analysis of ZPBI-MOF

**EDAX (Fig. 4)**

| Element | Line Type | Apparent Concentration | k Ratio | Wt% | Wt% Sigma | Atomic % | Standard Label | Factory Standard | Standard Calibration Date |
| --- | --- | --- | --- | --- | --- | --- | --- | --- | --- |
| C | K series | 1.15 | 0.01148 | 32.45 | 0.39 | 44.18 | C Vit | Yes |  |
| N | K series | 1.55 | 0.00275 | 12.32 | 0.56 | 14.38 | BN | Yes |  |
| O | K series | 3.82 | 0.01286 | 35.79 | 0.36 | 36.58 | SiO2 | Yes |  |
| Zn | L series | 1.70 | 0.01704 | 19.44 | 0.23 | 4.86 | Zn | Yes |  |
| Total: |  |  |  | 100.00 |  | 100.00 |  |  |  |

| Element | Line Type | Apparent Concentration | k Ratio | Wt% | Wt% Sigma | Atomic % | Standard Label | Factory Standard | Standard Calibration Date |
| --- | --- | --- | --- | --- | --- | --- | --- | --- | --- |
| C | K series | 1.18 | 0.01177 | 35.06 | 0.31 | 46.31 | C Vit | Yes |  |
| N | K series | 1.35 | 0.00241 | 12.69 | 0.45 | 14.38 | BN | Yes |  |
| O | K series | 3.23 | 0.01086 | 35.56 | 0.28 | 35.26 | SiO2 | Yes |  |
| Zn | L series | 1.28 | 0.01278 | 16.69 | 0.16 | 4.05 | Zn | Yes |  |
| Total: |  |  |  | 100.00 |  | 100.00 |  |  |  |

| red 88 | | | violet-14 | | blue 54 | | congo red | |
| --- | --- | --- | --- | --- | --- | --- | --- | --- |
| pH | dy removal | qe(mg/g) | dy removal | qe(mg/g) | dy removal | qe(mg/g) | dy removal | qe(mg/g) |
| 2 | 100 | 25 | 100 | 25 | 100 | 25 | 100 | 25 |
| 4 | 78.3 | 19.575 | 59 | 14.75 | 100 | 25 | 100 | 25 |
| 6 | 78.7 | 19.68 | 60.7 | 15.175 | 100 | 25 | 97.5 | 24.38 |
| 8 | 60 | 15 | 95.5 | 23.875 | 100 | 25 | 93.96 | 23.49 |

| red 88 | average | S.D |
| --- | --- | --- |
| 2 | 24.73333 | 0.46188 |
| 4 | 19.425 | 0.139194 |
| 6 | 19.54667 | 0.117189 |
| 8 | 14.8 | 0.264575 |
|  |  |  |
| violet-14 |  |  |
| 2 | 24.9 | 0.1 |
| 4 | 14.36667 | 0.375278 |
| 6 | 14.99167 | 0.187639 |
| 8 | 23.19167 | 0.687538 |
|  |  |  |
| blue 54 |  |  |
| 2 | 24.83333 | 0.152753 |
| 4 | 24.69667 | 0.300056 |
| 6 | 24.45667 | 0.505602 |
| 8 | 24.43333 | 0.51316 |
|  |  |  |
| congo red |  |  |
| 2 | 24.91333 | 0.141539 |
| 4 | 24.77 | 0.202237 |
| 6 | 24.29 | 0.147309 |
| 8 | 23.19667 | 0.258908 |


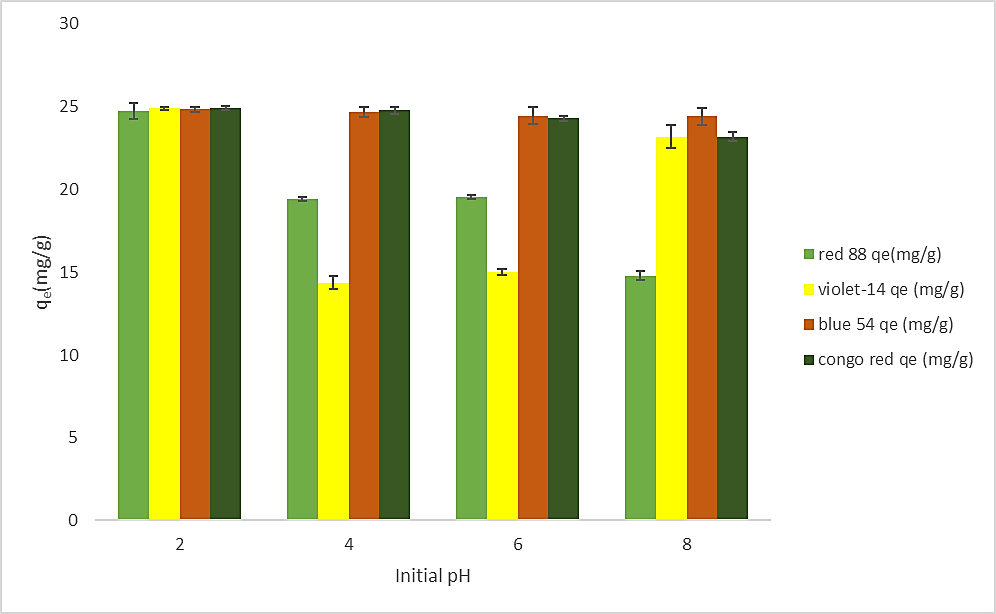


**Fig. 7.** Effect of pH value on dye adsorption (Initial dye concentration 20mg/L, Adsorbent mass 0.08g/L, Contact time 20 min, Tem. 25°C, Stirring 400 rpm)

|  | red 88 | | violet-14 | | blue 54 | | congo red | |
| --- | --- | --- | --- | --- | --- | --- | --- | --- |
| Ads | dy removal | qe(mg/g) | dy removal | qe(mg/g) | dy removal | qe(mg/g) | dy removal | qe(mg/g) |
| 1 | 100 | 200 | 91 | 182 | 86.8 | 173.6 | 85.87 | 171.75 |
| 2 | 100 | 100 | 97 | 97.10 | 95 | 95.00 | 90 | 89.70 |
| 6 | 100 | 33.33 | 100 | 33.33 | 100 | 33.33 | 97.50 | 32.5 |
| 8 | 100 | 25 | 100 | 25 | 100 | 25 | 100 | 25 |
| 10 | 100 | 20 | 100 | 20 | 100 | 20 | 100 | 20 |

| red 88 | average | S.D |
| --- | --- | --- |
| 1 | 199.8333 | 0.288675 |
| 2 | 99.6 | 0.52915 |
| 6 | 33.21 | 0.115326 |
| 8 | 24.95 | 0.086603 |
| 10 | 19.5 | 0.5 |
|  |  |  |
| violet-14 |  |  |
| 1 | 181.5 | 0.5 |
| 2 | 96.53333 | 0.550757 |
| 6 | 33.14333 | 0.169214 |
| 8 | 24.43333 | 0.51316 |
| 10 | 19.6 | 0.4 |
|  |  |  |
| blue 54 |  |  |
| 1 | 172.8667 | 0.80829 |
| 2 | 94.53333 | 0.503322 |
| 6 | 33.10667 | 0.193477 |
| 8 | 24.78333 | 0.202073 |
| 10 | 19.8 | 0.264575 |
|  |  |  |
| congo red |  |  |
| 1 | 170.7833 | 0.889288 |
| 2 | 89.41677 | 0.368556 |
| 6 | 32.21667 | 0.25658 |
| 8 | 24.67667 | 0.460036 |
| 10 | 19.95 | 0.05 |


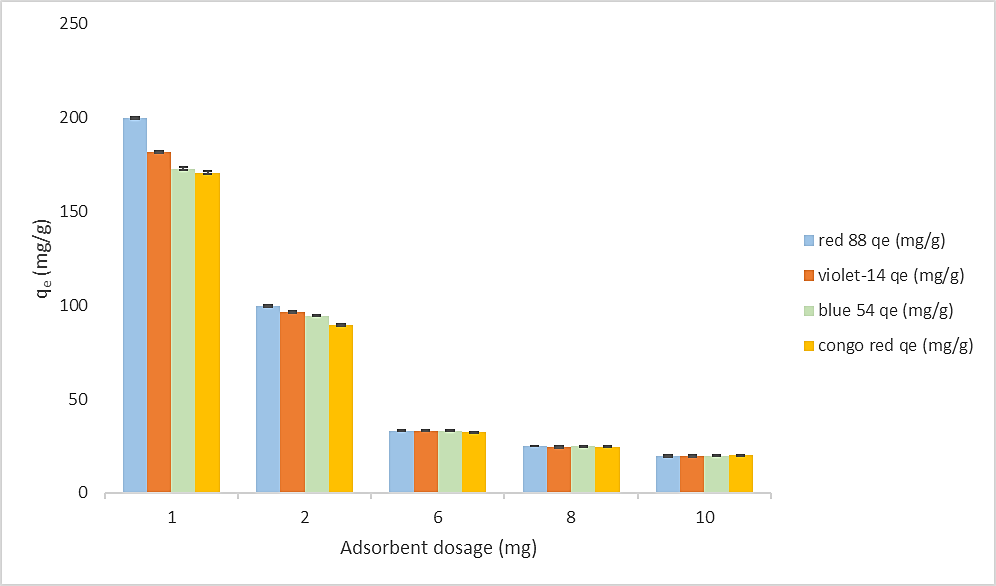


**Fig. 8.** Effect of adsorbent dose (1- 10 mg) on dye adsorption (Initial dye concentration 20mg/l, Adsorbent

mass 0.08 g/L, Contact time 20 min, Tem. 25-65ºC).

|  | red 88 | | violet-14 | | blue 54 | | congo red | |
| --- | --- | --- | --- | --- | --- | --- | --- | --- |
| Tem | dye removal | qe (mg/g) | dye removal | qe(mg/g) | dye removal | qe(mg/g) | dye removal | qe(mg/g) |
| 25 | 100 | 25.000 | 100.00 | 25.000 | 100 | 25.000 | 100 | 25.000 |
| 35 | 80.25 | 20.15 | 90.52 | 22.63 | 99.76 | 24.94 | 99.04 | 24.76 |
| 45 | 79.64 | 19.911 | 89.2 | 22.3 | 99.64 | 24.91 | 98.96 | 24.74 |
| 65 | 100 | 25 | 87.2 | 21.8 | 98.04 | 24.51 | 98.48 | 24.62 |

| red 88 | average | S.D |
| --- | --- | --- |
| 25 | 24.5 | 0.5 |
| 35 | 20 | 0.15 |
| 45 | 19.287 | 0.540978 |
| 65 | 24.86333 | 0.228108 |
|  |  |  |
| violet-14 |  |  |
| 25 | 24.43333 | 0.51316 |
| 35 | 21.87667 | 0.821969 |
| 45 | 21.93333 | 0.404145 |
| 65 | 21 | 0.916515 |
|  |  |  |
| blue 54 |  |  |
| 25 | 24.85 | 0.217945 |
| 35 | 24.57 | 0.500899 |
| 45 | 24.49 | 0.459021 |
| 65 | 24.30333 | 0.20502 |
|  |  |  |
| congo red |  |  |
| 25 | 24.44 | 0.510686 |
| 35 | 24.29667 | 0.406489 |
| 45 | 24.28667 | 0.397157 |
| 65 | 24.47333 | 0.236925 |


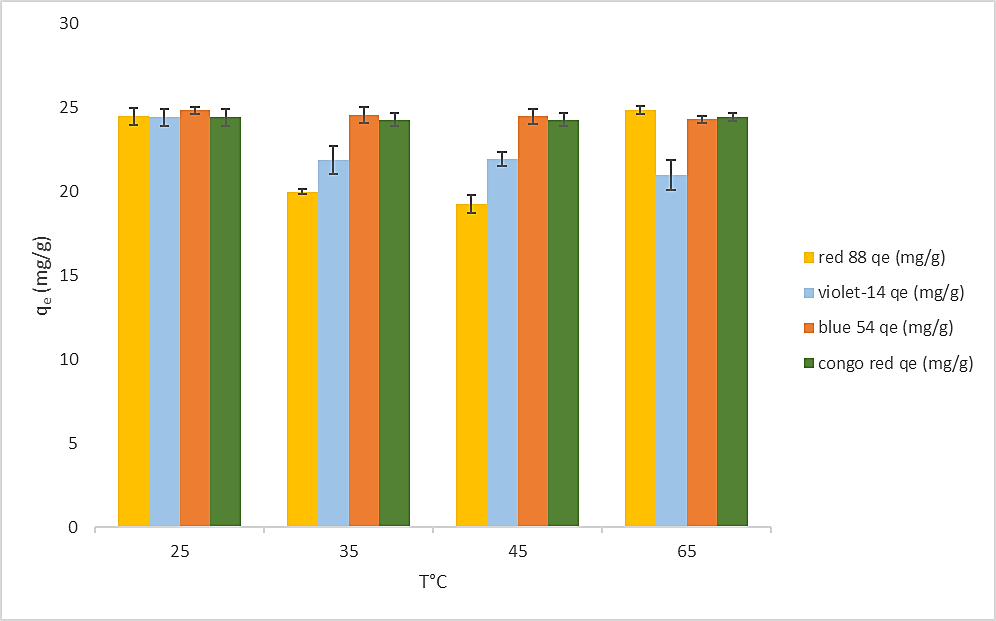


**Fig. 9.** Effect of temperature (25-65 ºC) on dye adsorption

(Initial dye concentration 20mg/L, Adsorbent mass 0.08g/L, Contact time 20 min, Stirring 400 rpm).

|  | red 88 | | violet-14 | | blue 54 | | congo red | |
| --- | --- | --- | --- | --- | --- | --- | --- | --- |
| Con | dy removal | qe(mg/g) | dy removal | qe(mg/g) | dy removal | qe(mg/g) | dy removal | qe(mg/g) |
| 10 | 100 | 12.5 | 100 | 12.5 | 100 | 12.5 | 100 | 12.5 |
| 20 | 100 | 25 | 100 | 25 | 100 | 25 | 100 | 25 |
| 30 | 100 | 37.5000 | 100 | 37.5000 | 100 | 37.5000 | 100 | 37.5000 |
| 50 | 99 | 61.875 | 99.48 | 62.18 | 95 | 59.375 | 99.85 | 62.41 |
| 100 | 99 | 123.75 | 99.5 | 124.375 | 95 | 118.75 | 99.8 | 124.75 |
| 150 | 99.66 | 185.000 | 98.99 | 185.610 | 93.33 | 175.000 | 99.83 | 187.188 |
| 200 | 98.48 | 246.21 | 99.15 | 247.88 | 94.44 | 236.1125 | 99.80 | 249.5 |
| 250 | 98.41 | 307.55 | 99.29 | 310.3 | 90.00 | 281.25 | 99.80 | 311.875 |
| 300 | 98.66 | 369.975 | 98.98 | 371.2 | 90.99 | 341.225 | 99.80 | 374.273 |
| 350 | 98.85 | 432.5 | 99.38 | 434.83 | 92.06 | 402.78 | 99.79 | 436.6 |
| 400 | 98.98 | 494.9437 | 99.66 | 498.3 | 93.79 | 468.96 | 99.81 | 499.06 |
| 500 | 99.212 | 620.075 | 99.56 | 622.25 | 95.52 | 597.03 | 99.83 | 623.96 |
| 600 | 99.33 | 744.9975 | 99.58 | 746.9 | 93.92 | 707.47 | 99.84 | 748.85 |
| 800 | 87.5 | 875 | 85.00 | 850 | 87.76 | 877.68 | 87.50 | 875 |

| red 88 | average | S.D |
| --- | --- | --- |
| 10 | 12.08333 | 0.381881 |
| 20 | 24.85 | 0.155242 |
| 30 | 37.2 | 0.264575 |
| 50 | 61.54833 | 0.477764 |
| 100 | 123.1267 | 0.977565 |
| 150 | 184.7867 | 0.369504 |
| 200 | 246.1367 | 0.118462 |
| 250 | 307.3333 | 0.189297 |
| 300 | 369.7583 | 0.274241 |
| 350 | 431.8333 | 0.763763 |
| 400 | 493.4146 | 1.47519 |
| 500 | 619.8583 | 0.312583 |
| 600 | 742.6658 | 2.080265 |
| 800 | 872.44 | 2.370823 |
|  |  |  |
| violet-14 |  |  |
| 10 | 12.23333 | 0.251661 |
| 20 | 24.51667 | 0.671441 |
| 30 | 37.23333 | 0.251661 |
| 50 | 61.17 | 0.976883 |
| 100 | 123.985 | 0.397712 |
| 150 | 185.1633 | 0.563678 |
| 200 | 247.7467 | 0.166533 |
| 250 | 309.8 | 0.7 |
| 300 | 370.7667 | 0.665833 |
| 350 | 431.3433 | 3.241918 |
| 400 | 495.4333 | 3.187998 |
| 500 | 621.2667 | 1.000417 |
| 600 | 744.7667 | 1.890326 |
| 800 | 848.4333 | 2.713546 |
|  |  |  |
| blue 54 |  |  |
| 10 | 12.02 | 0.470319 |
| 20 | 24.83333 | 0.271539 |
| 30 | 37.19667 | 0.305014 |
| 50 | 59.20833 | 0.190941 |
| 100 | 118.6167 | 0.266333 |
| 150 | 174.2267 | 0.688573 |
| 200 | 235.0775 | 0.998508 |
| 250 | 280.1833 | 0.987843 |
| 300 | 340.7117 | 0.550689 |
| 350 | 402.51 | 0.441928 |
| 400 | 468.5367 | 0.489932 |
| 500 | 596.6767 | 0.586202 |
| 600 | 706.8233 | 0.750755 |
| 800 | 877.2233 | 0.580546 |
|  |  |  |
| congo red |  |  |
| 10 | 11.91 | 0.639766 |
| 20 | 24.76667 | 0.225462 |
| 30 | 36.78667 | 0.752684 |
| 50 | 62.01 | 0.675574 |
| 100 | 123.9167 | 0.877971 |
| 150 | 187.1125 | 0.099216 |
| 200 | 247.8333 | 2.466441 |
| 250 | 311.0417 | 0.813301 |
| 300 | 373.1977 | 1.90338 |
| 350 | 434.8667 | 1.8037 |
| 400 | 492.8533 | 5.393935 |
| 500 | 621.2367 | 2.515558 |
| 600 | 744.6167 | 4.437435 |
| 800 | 872.7333 | 2.411086 |


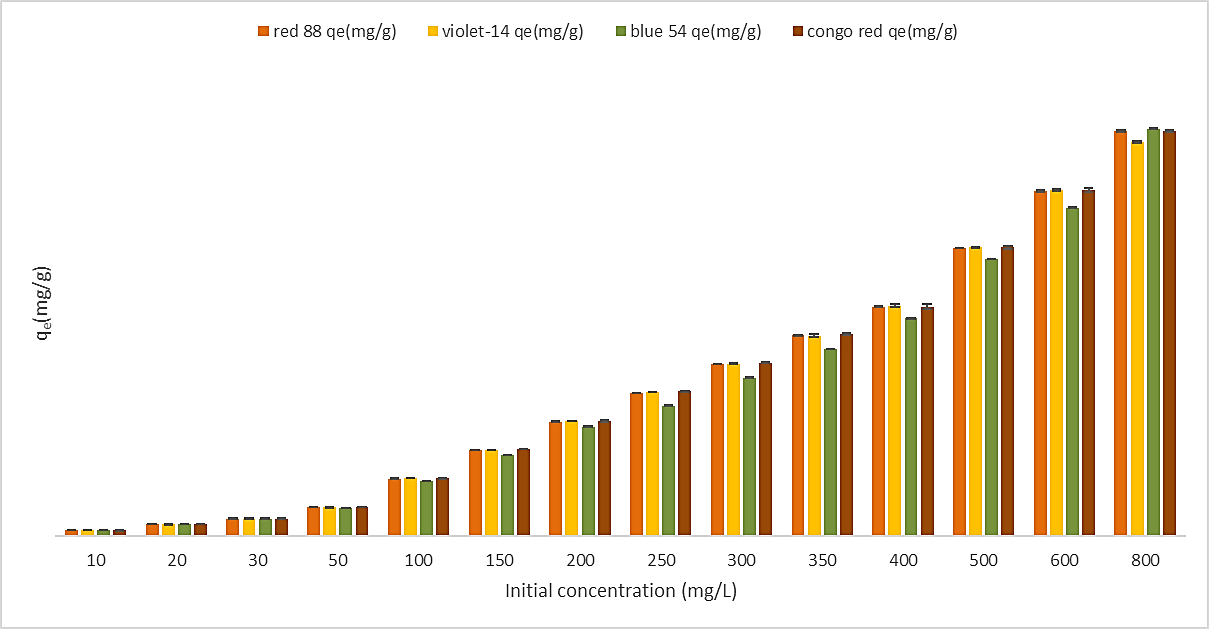


**Fig. 10.** Effect of initial concentration (10-800 mg/L) on dye adsorption (Adsorbent mass 0.08 g/L, Contact time 20 min, Tem. 25-65ºC).

| red 88 | | violet-14 | | blue 54 | | congo red | |
| --- | --- | --- | --- | --- | --- | --- | --- |
| 50 | 250 | 50 | 250 | 50 | 250 | 50 | 250 |
| 2 | 0.0161 | 3.9 | 0.016 | 0.4 | 0.0168 | 13.88 | 0.016 |
| 1 | 0.008 | 2 | 0.008 | 0.2 | 0.0084 | 5 | 0.00804 |
| 0.5 | 0.0054 | 0.66 | 0.0053 | 0.1 | 0.00571 | 4 | 0.0053 |
| 0.32 | 0.004 | 0.589 | 0.00403 | 0.09 | 0.00423 | 2.5 | 0.004 |
| 0.25 | 0.0032 | 0.568 | 0.00322 | 0.04 | 0.0044 | 2 | 0.0032 |
| 0.24 | 0.0027 | 0.328 | 0.00269 | 0.037 | 0.0029 | 1.72 | 0.0022 |
| 0.25 | 0.0023 | 0.468 | 0.00229 | 0.036 | 0.00248 | 1.38 | 0.00229 |
| 0.24 | 0.002 | 0.735 | 0.002006 | 0.0402 | 0.00213 | 1.32 | 0.002003 |
| 0.25 | 0.0016 | 0.4545 | 0.0016 | 0.044 | 0.00167 | 1.2 | 0.0016 |
| 0.24 | 0.0013 | 0.4032 | 0.00133 | 0.027 | 0.001413 | 1.08 | 0.00133 |
| 0.01 | 0.001 | 0.00833 | 0.001176 | 0.0102 | 0.001139 | 0.01 | 0.0011 |


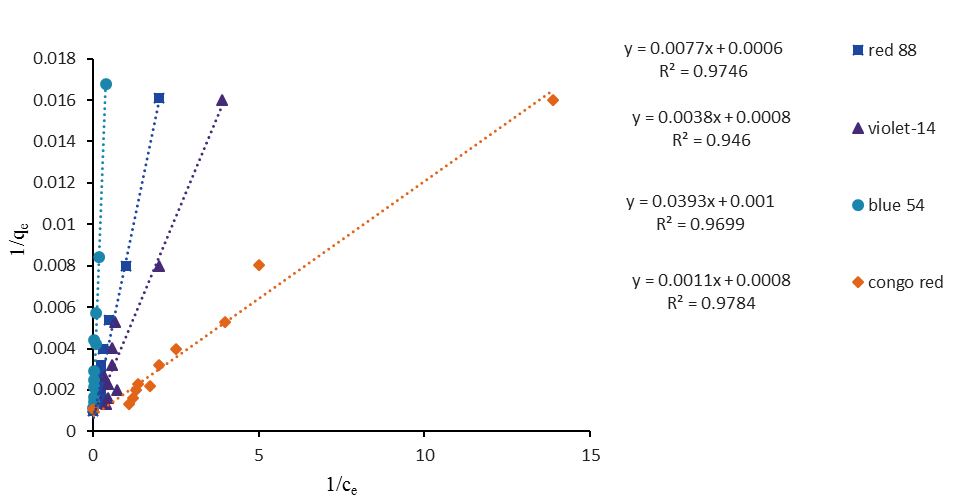


**Fig. 11.** Linearized Langmuir isotherm for the adsorption of dyes by ZPBIF-1 composites.

| red 88 50 | | red 88 250 | | violet-14 50 | | violet-14 250 | |
| --- | --- | --- | --- | --- | --- | --- | --- |
| 0 | 0 | 0 | 0 | 0 | 0 | 0 | 0 |
| 10 | 0.193 | 10 | 0.00339 | 10.00 | 0.178 | 10 | 0.0322 |
| 15 | 0.282 | 15 | 0.0487 | 15.00 | 0.265 | 15 | 0.0483 |
| 20 | 0.323 | 20 | 0.06503 | 20.00 | 0.321 | 20 | 0.0644 |


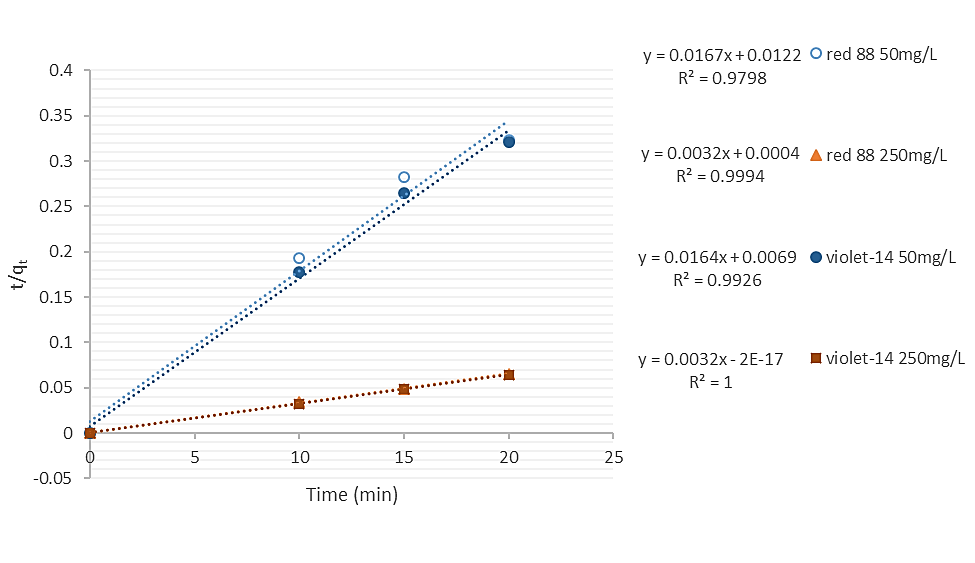


| blue 54 50 | | blue 54 250 | | congo red 50 | | congo red 250 | |
| --- | --- | --- | --- | --- | --- | --- | --- |
| 0 | 0 | 0 | 0 | 0 | 0 | 0 | 0 |
| 10 | 0.178 | 10 | 0.036 | 10 | 0.162 | 10 | 0.03209 |
| 15 | 0.265 | 15 | 0.054 | 15 | 0.2408 | 15 | 0.04808 |
| 20 | 0.336 | 20 | 0.0711 | 20 | 0.32 | 20 | 0.0641 |


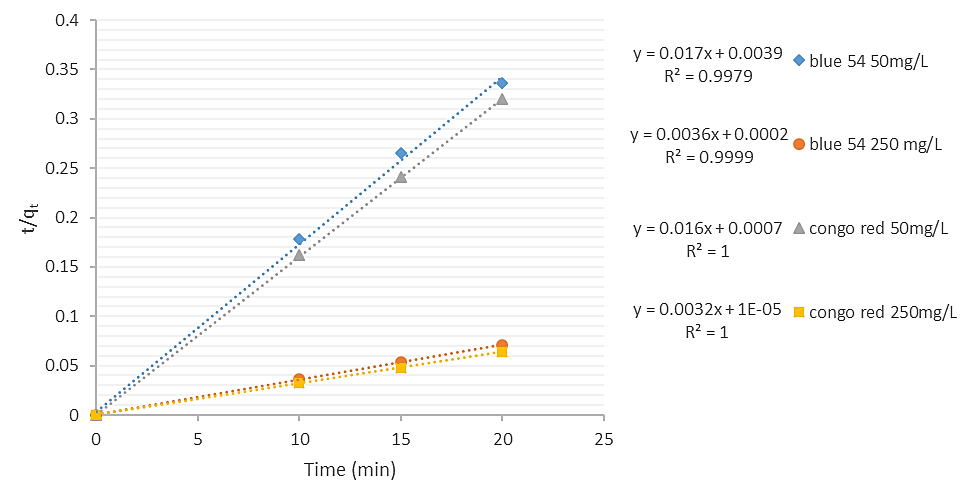


**Fig. 12.** Kinetics plot for the adsorption of dyes by ZPBIF-1. The pseudo- second-order model (Contact time=20 min, Tem=25-65°C, Adsorbent mass=0. 08g/L, Initial concertation 50 and 250mg/L, Stirring=400 rpm and pH=2)

| red 88 | | | violet-14 | | | Congo red | | |
| --- | --- | --- | --- | --- | --- | --- | --- | --- |
|  | C_e_ | Dye removal |  | C_e_ | Dye removal |  | C_e_ | Dye removal |
| 1 | 0 | 100 | 1 | 0 | 100 | 1 | 0 | 100 |
| 2 | 10 | 90 | 2 | 20 | 80 | 2 | 10 | 90 |
| 3 | 20 | 80 | 3 | 30 | 70 | 3 | 13 | 87 |

| red 88 | average | SD |
| --- | --- | --- |
| 1 | 99.666667 | 0.5773503 |
| 2 | 89.833333 | 0.2886751 |
| 3 | 79.653333 | 0.3008876 |
|  |  |  |
| violet-14 |  |  |
| 1 | 99.666667 | 0.5773503 |
| 2 | 79.533333 | 0.5033223 |
| 3 | 69.5 | 0.5 |
|  |  |  |
| congo red |  |  |
| 1 | 100 | 0 |
| 2 | 89.476667 | 0.5016307 |
| 3 | 86.166667 | 0.7637626 |

**
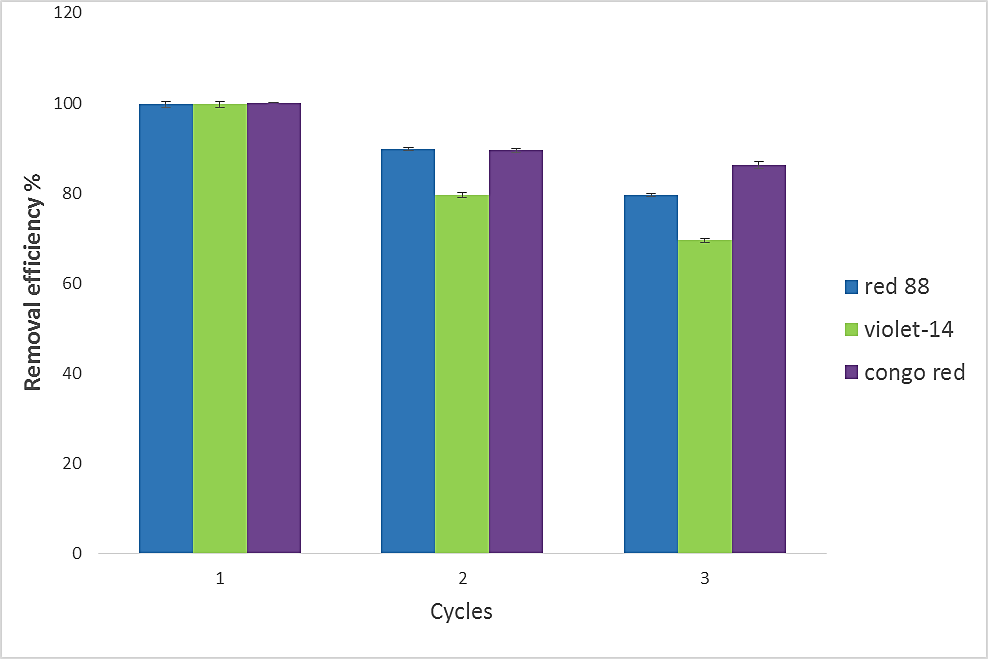
**

**Fig. 15.**  Recyclability study of ZPBIF-1 towards dyes
