# Supplementary material for: Design, synthesis, and characterization of a novel Zn(II)-2-phenyl benzimidazole framework for the removal of organic dyes
Source: Sci Rep. 2022 Jul 20;12:12431. doi: 10.1038/s41598-022-16753-8 (PMC9300708; doi:10.1038/s41598-022-16753-8)
Supplement: Supplementary file 2 — Supplementary Information 2. [file 41598_2022_16753_MOESM2_ESM.docx]

**Novel Zn (II)-2-phenyl benzimidazole framework (ZPBIF-1) for the efficient removal of organic dyes: synthesis, characterization, adsorption, kinetic, and thermodynamic studies**

Shabnam Alibakhshi^1^, Ashraf S. Shahvelayati^a1^, Shabnam Sheshmani^1^, Maryam Ranjbar^2^, Saeid Souzangarzadeh^1^

*^1^Department of Chemistry, College of Basic Sciences, Yadegar-e- Imam Khomeini (RAH) Shahre Ray Branch, Islamic Azad University, Tehran, Iran.* * Corresponding Author: [avelayati@yahoo.com](mailto:avelayati@yahoo.com), [a_shahvelayati@iausr.ac.ir](mailto:a_shahvelayati@iausr.ac.ir)

*^2^Department of Chemical Technologies, Iranian Research Organization for Science and Technology (IROST), Tehran, Iran*

**Supplementary file 2: The raw data for tables 1, 3, 4, 5**

| red 88 | | violet-14 | | blue 54 | | congo red | |
| --- | --- | --- | --- | --- | --- | --- | --- |
| -0.3 | 1.791 | -0.602 | 1.79 | 0.397 | 1.773 | -1.14 | 1.79 |
| 0 | 2.092 | -0.30 | 2.094 | 0.69 | 2.074 | -0.69 | 2.09 |
| 0.3 | 2.267 | 0.18 | 2.268 | 1.00 | 2.24 | -0.60 | 2.27 |
| 0.48 | 2.391 | 0.23 | 2.394 | 1.04 | 2.37 | -0.39 | 2.39 |
| 0.60 | 2.487 | 0.25 | 2.491 | 1.39 | 2.44 | -0.30 | 2.49 |
| 0.60 | 2.568 | 0.48 | 2.569 | 1.43 | 2.53 | -0.23 | 2.64 |
| 0.60 | 2.635 | 0.32 | 2.638 | 1.44 | 2.6 | -0.14 | 2.64 |
| 0.61 | 2.694 | 0.13 | 2.697 | 1.39 | 2.67 | -0.12 | 2.69 |
| 0.60 | 2.792 | 0.34 | 2.793 | 1.34 | 2.77 | -0.08 | 2.79 |
| 0.60 | 2.872 | 0.39 | 2.873 | 1.56 | 2.84 | -0.04 | 2.87 |
| 2 | 2.942 | 2.08 | 2.929 | 1.99 | 2.94 | 2.08 | 2.92 |


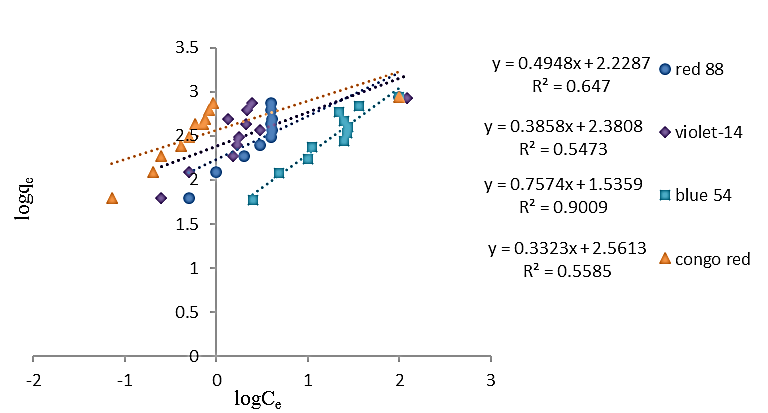
.

Linearized Frundlich isotherm for the adsorption of dyes by ZPBIF-1 composites.

| red 88 | | violet-14 | | blue 54 | | congo red | |
| --- | --- | --- | --- | --- | --- | --- | --- |
| -0.6931 | 61.875 | -1.3600 | 62.18 | 0.9160 | 59.375 | -2.6310 | 62.41 |
| 0.0000 | 123.75 | -0.6931 | 124.375 | 1.6000 | 118.75 | -1.6094 | 124.375 |
| 0.9631 | 185 | 0.4130 | 185.61 | 2.3000 | 175 | -1.3862 | 187.1875 |
| 1.11 | 246.21 | 0.52 | 247.88 | 2.40 | 236.1125 | -0.92 | 249.5 |
| 1.37 | 307.55 | 0.56 | 310.3 | 3.21 | 281.25 | -0.69 | 311.875 |
| 1.390 | 369.975 | 1.110 | 371.2 | 3.290 | 341.225 | -0.544 | 436.775 |
| 1.3800 | 432.5 | 0.7500 | 434.83 | 3.3200 | 402.78 | -0.3280 | 436.6 |
| 1.3970 | 494.9437 | 0.3070 | 498.3 | 3.2100 | 468.96 | -0.2850 | 499.06 |
| 1.3711 | 620.075 | 0.788 | 622.25 | 3.1 | 597.03 | -0.18 | 623.96 |
| 1.386 | 744.9975 | 0.908 | 746.9 | 3.59 | 707.47 | -0.083 | 748.85 |
| 4.6 | 875 | 4.787 | 850 | 4.58 | 877.68 | 4.6 | 875 |


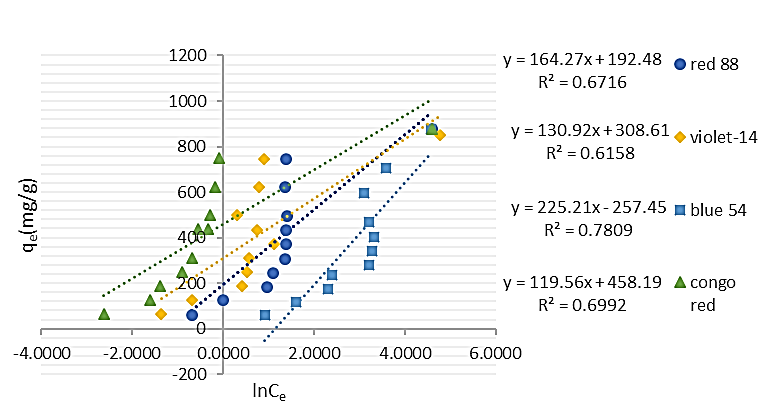


.Linearized Tempkin isotherm for the adsorption of dyes by ZPBIF-1 composites


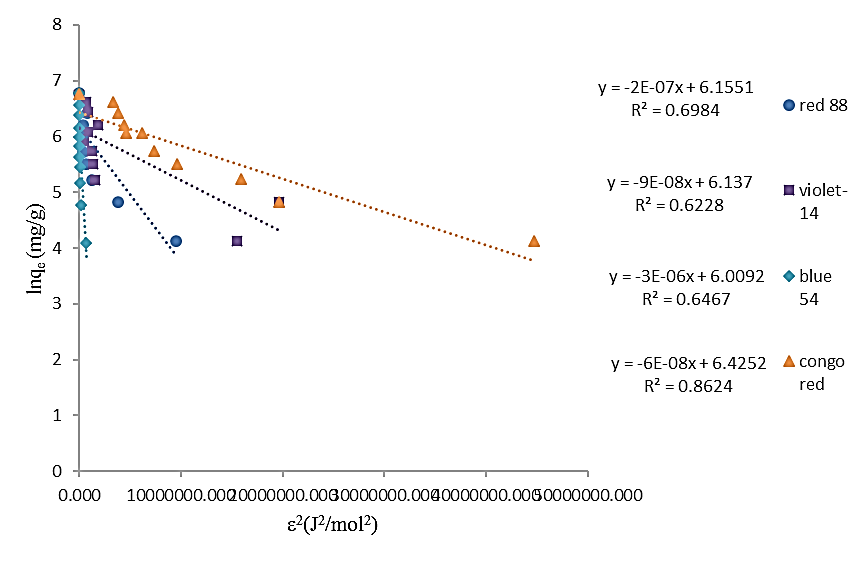


| red 88 | | violet-14 | |
| --- | --- | --- | --- |
| 9521916.200 | 4.1251 | 15513412.100 | 4.13 |
| 3790411.5900 | 4.8182 | 19687655.1000 | 4.82 |
| 1297007.3200 | 5.2203 | 1580361.5800 | 5.223 |
| 640984.1000 | 5.5061 | 1317430.7200 | 5.512 |
| 399967.35000 | 5.7286 | 1241363.96000 | 5.737 |
| 389332.26 | 5.9134 | 495969.07 | 5.916 |
| 392829.5300 | 6.0695 | 904329.7920 | 6.074 |
| 403609.6500 | 6.2044 | 1863016.1600 | 6.211 |
| 807219.3000 | 6.4298 | 860967.5350 | 6.433 |
| 392477.6800 | 6.6133 | 703807.738 | 6.615 |
| 781.106 | 6.774 | 422.34 | 6.745 |

|  |  |  |  |
| --- | --- | --- | --- |
| blue 54 | | congo red | |
| 694277.427 | 4.083 | 44725993.600 | 4.13 |
| 203849.9500 | 4.77 | 19687655.1000 | 4.82 |
| 55707.4300 | 5.164 | 15884846.8000 | 5.23 |
| 45552.0110 | 5.46 | 9624371.7600 | 5.51 |
| 9433.34100 | 5.63 | 7401563.70000 | 5.74 |
| 8099.04 | 5.832 | 6158880.65 | 6.07 |
| 7674.9144 | 5.99 | 4650500.5700 | 6.07 |
| 9558.9110 | 6.15 | 4386784.1400 | 6.21 |
| 11722.2970 | 6.391 | 3820788.6000 | 6.43 |
| 4498.5250 | 6.561 | 3339812.6800 | 6.61 |
| 633.9200 | 6.777 | 6017.1600 | 6.77 |
|  |  |  |  |

.Linearized Dubinin–Radushkevich (D-R) isotherm for dye adsorption by ZPBIF-1 composites.

**Table 1** Linearized isotherm coefficients for dye adsorption (Initial dye concentration 50-800 mg/L, Adsorbent mass 0.08 g/L, Contact time 20 min, Tem. 25-65° C).

| Isotherm Models | Isotherm parameters | Acid red 88 | Basic Violet 14 | Basic Blue 54 | Congo red |
| --- | --- | --- | --- | --- | --- |
| Langmuir model | Q_m_(mg /g)  K_L_ (L /mg)  R^2^ | 1666.66  0.077  0.9746 | 1250  0.210  0.946 | 1000  0.025  0 .9699 | 1250  0.727  0.9784 |
| Freundlich model | K_f_ (mg/g)  n  R^2^ | 169.31  2.02  0.6470 | 240.32  2.59  0.5473 | 34.34  1.32  0.9009 | 364.16  3.00  0.5585 |
| Tempkin model | K_T_ (L/mg)  b_T_ (J/mol)  R^2^  B_1_ | 3.22  17.09  0.6716  164.27 | 10.56  18.91  0.6158  130.92 | 3.13  10.99  0.7809  225.21 | 46.17  20.71  0.6992  119.56 |
| Dubinin- Radushkevich model | Q_s_ (mg/ g)  K_ad_ (mol^2^/J^2^)  E (kJ/ mol)  R^2^ | 471.11  0.0000007  0.845  0.6784 | 462.66  0.00000009  2.357  0.6228 | 407.15  0.000003  0.408  0.6467 | 617.20  0.00000006  2.886  0.8624 |
| Separation factor (R_L_) | C_0_ (mg/L) 50-800 | 0.206-0.015 | 0.086-0.005 | 0.444-0.047 | 0.026-0.001 |

| red 88 50 | | red 88 250 | | violet-14 50 | | violet-14 250 | |
| --- | --- | --- | --- | --- | --- | --- | --- |
| 0 | 3.91 | 0 | 5.521 | 0 | 3.91 | 0 | 5.521 |
| 10 | 2.16 | 10 | 2.6755 | 10.00 | 1.6639 | 10 | 0.587 |
| 15 | 2.01 | 15 | 1.38 | 15.00 | 1.55 | 15 | 0.5766 |
| 20 | -0.69 | 20 | 1.3686 | 20.00 | -1.36 | 20 | 0.565 |

**
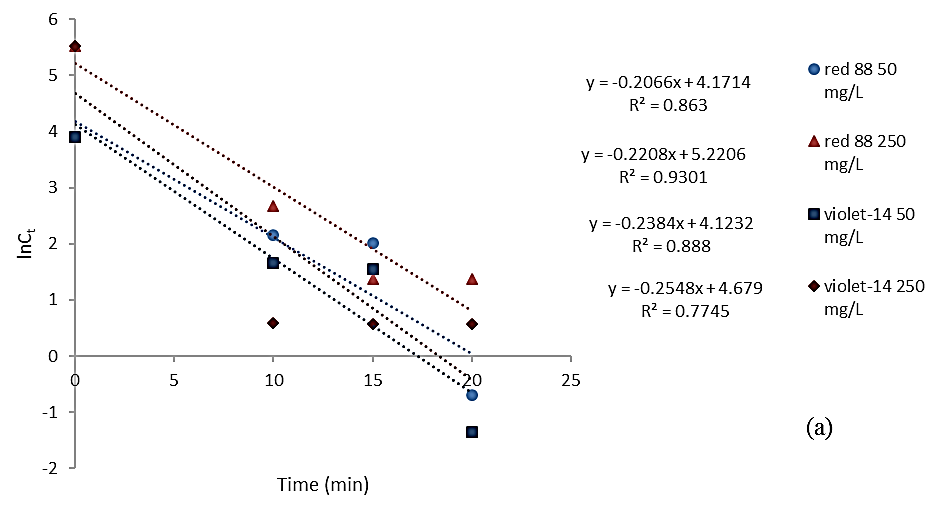
**

| Blue 54 50 | | Blue 54 250 | | Congo red 50 | | Congo red 250 | |
| --- | --- | --- | --- | --- | --- | --- | --- |
| 0 | 3.91 | 0 | 5.521 | 0 | 3.91 | 0 | 5.521 |
| 10 | 1.39 | 10 | 3.47 | 10.00 | -0.253 | 10 | -0.253 |
| 15 | 0.99 | 15 | 3.32 | 15.00 | -1.783 | 15 | -1.783 |
| 20 | 0.916 | 20 | 3.21 | 20.00 | -2.748 | 20 | -2.748 |

**
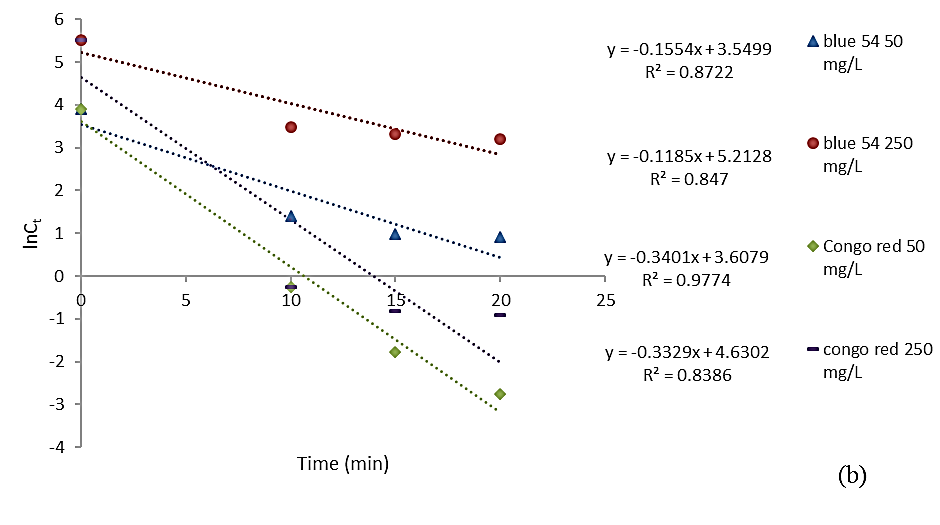
**

| red 88 50 | | red 88 250 | | violet-14 50 | | violet-142 50 | |
| --- | --- | --- | --- | --- | --- | --- | --- |
| 0 | 4.125 | 0 | 5.7286 | 0 | 4.13 | 0 | 5.737 |
| 10 | 2.324 | 10 | 2.5802 | 10.00 | 1.83 | 10 | -2.99 |
| 15 | 2.167 | 15 | -0.2995 | 15.00 | 1.723 | 15 | -3.688 |

**
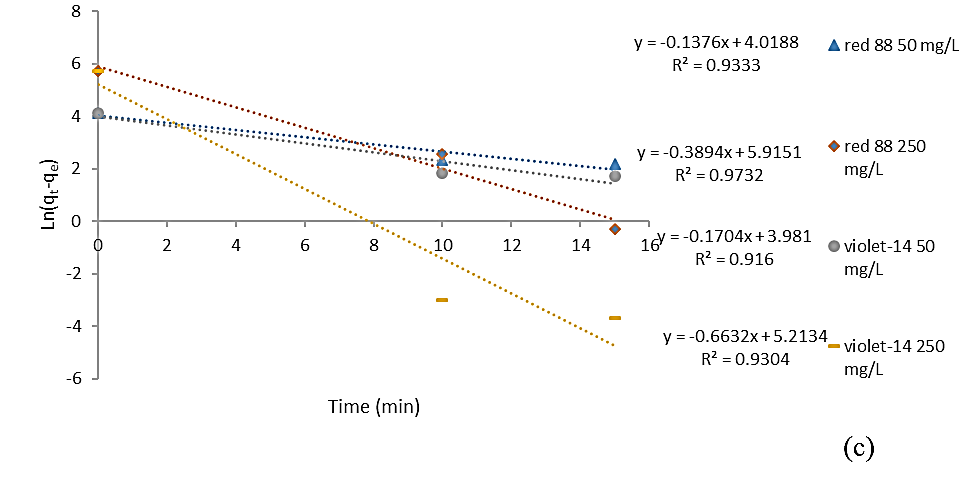
**

| Blue 54 50 | | Blue 54 250 | | Congo red 50 | | Congo red 250 | |
| --- | --- | --- | --- | --- | --- | --- | --- |
| 0 | 4.083 | 0 | 5.639 | 0 | 4.133 | 0 | 5.743 |
| 10 | 0.64 | 10 | 2.207 | 10.00 | -0.127 | 10 | -0.75 |
| 15 | -1.38 | 15 | 1.25 | 15.00 | -1.897 | 15 | -2.99 |

**
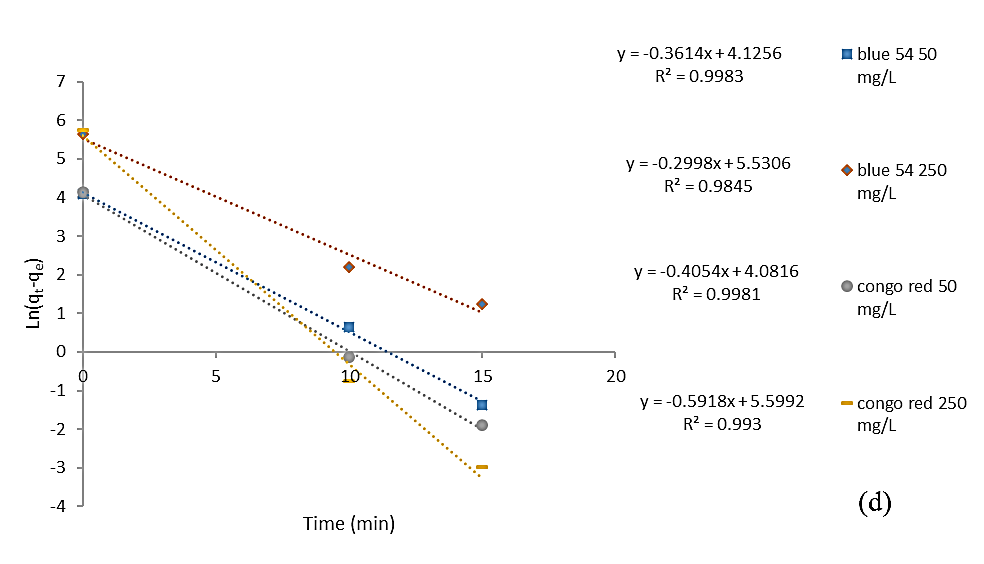
**

Kinetics plot for the adsorption of dyes by ZPBIF-1.

The first –order model (a-b), and pseudo–first –order model (c-d) (Contact time=20 min, Tem=25-65°C, Adsorbent mass=0. 08g/L,Initial concertation 50 and 250mg/L, Stirring=400 rpm, and pH=2).

| red 88 50 | | red 88 250 | | violet-14 50 | | violet-14 250 | |
| --- | --- | --- | --- | --- | --- | --- | --- |
| 0 | 0.02 | 0 | 0.004 | 0 | 0.02 | 0 | 0.004 |
| 10 | 0.1152 | 10 | 0.0688 | 10.00 | 0.189 | 10 | 0.555 |
| 15 | 0.13351 | 15 | 0.25 | 15.00 | 0.21 | 15 | 0.561 |
| 20 | 2 | 20 | 0.252 | 20.00 | 3.9 | 20 | 0.568 |


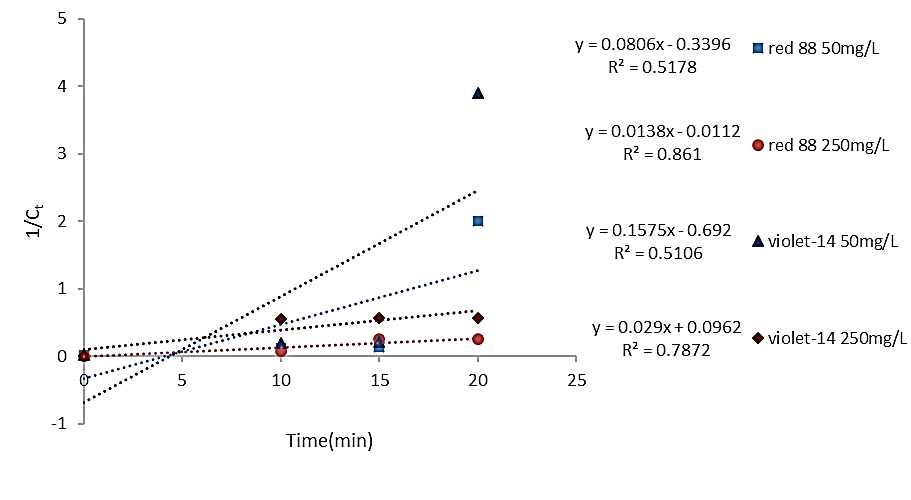


| Blue 54 50 | | Blue 54 250 | | Congo red 50 | | Congo red 250 | |
| --- | --- | --- | --- | --- | --- | --- | --- |
| 0 | 0.02 | 0 | 0.004 | 0 | 0.02 | 0 | 0.004 |
| 10 | 0.248 | 10 | 0.0309 | 10.00 | 1.28 | 10 | 1.28 |
| 15 | 0.37 | 15 | 0.035 | 15.00 | 5.95 | 15 | 2.27 |
| 20 | 0.4 | 20 | 0.04 | 20.00 | 15.62 | 20 | 2.5 |


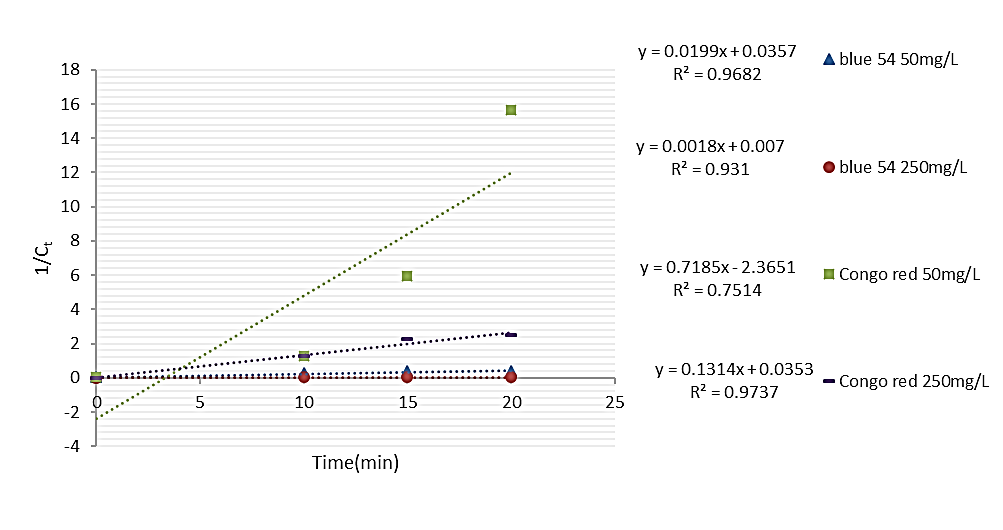


Kinetics plot for the adsorption of dyes by ZPBIF-1.

The and second-order model (Contact time=20 min, Tem=25-65°C, Adsorbent mass=0. 08g/L,Initial concertation 50 and 250mg/L, Stirring=400 rpm and pH=2).

| red 88 50 | | red 88 250 | | violet-14 50 | | violet-14 250 | |
| --- | --- | --- | --- | --- | --- | --- | --- |
| 3.16 | 51.65 | 3.16 | 294.35 | 3.16 | 55.9 | 3.16 | 310.25 |
| 3.87 | 53.1375 | 3.87 | 307.5 | 3.87 | 56.575 | 3.87 | 310.275 |
| 4.47 | 61.875 | 4.47 | 307.55 | 4.47 | 62.18 | 4.47 | 310.3 |


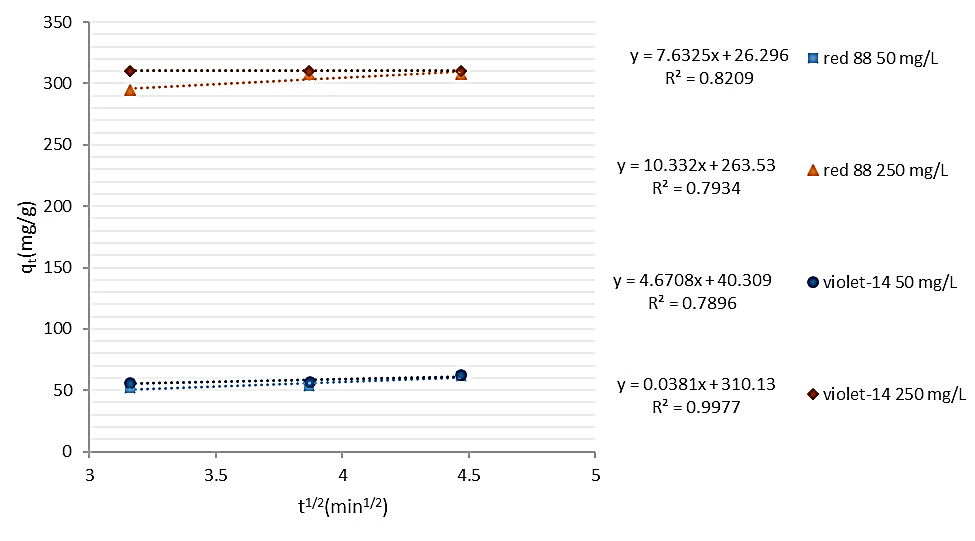


| Blue 54 50 | | Blue 54 250 | | Congo red 50 | | Congo red 250 | |
| --- | --- | --- | --- | --- | --- | --- | --- |
| 3.16 | 57.465 | 3.16 | 272.16 | 3.16 | 61.53 | 3.16 | 311.53 |
| 3.87 | 59.125 | 3.87 | 277.75 | 3.87 | 62.29 | 3.87 | 311.95 |
| 4.47 | 59.375 | 4.47 | 281.25 | 4.47 | 62.41 | 4.47 | 312 |


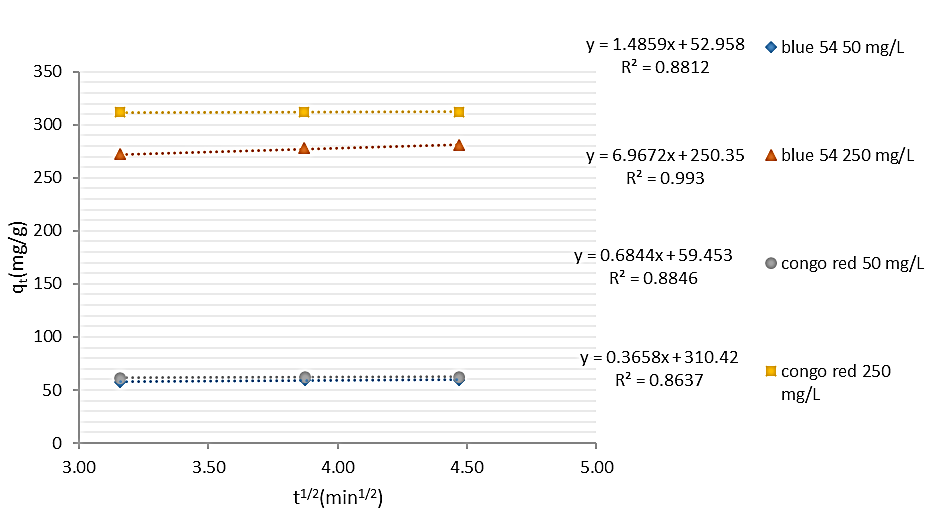


Kinetics plot for the adsorption of dyes by ZPBIF-1. The intraparticle diffusion model (Contact time=20 min, Tem=25-65°C, Adsorbent mass=0. 08g/L,Initial concertation 50 and 250mg/L, Stirring=400 rpm, and pH=2).

| red 88 50 | | red 88 250 | | violet-14 50 | | violet-14 250 | |
| --- | --- | --- | --- | --- | --- | --- | --- |
| 2.3 | 51.65 | 2.3 | 294.35 | 2.3 | 55.9 | 2.3 | 310.25 |
| 2.7 | 53.1375 | 2.7 | 307.5 | 2.7 | 56.575 | 2.7 | 310.275 |
| 2.99 | 61.875 | 2.99 | 307.55 | 2.99 | 62.18 | 2.99 | 310.3 |


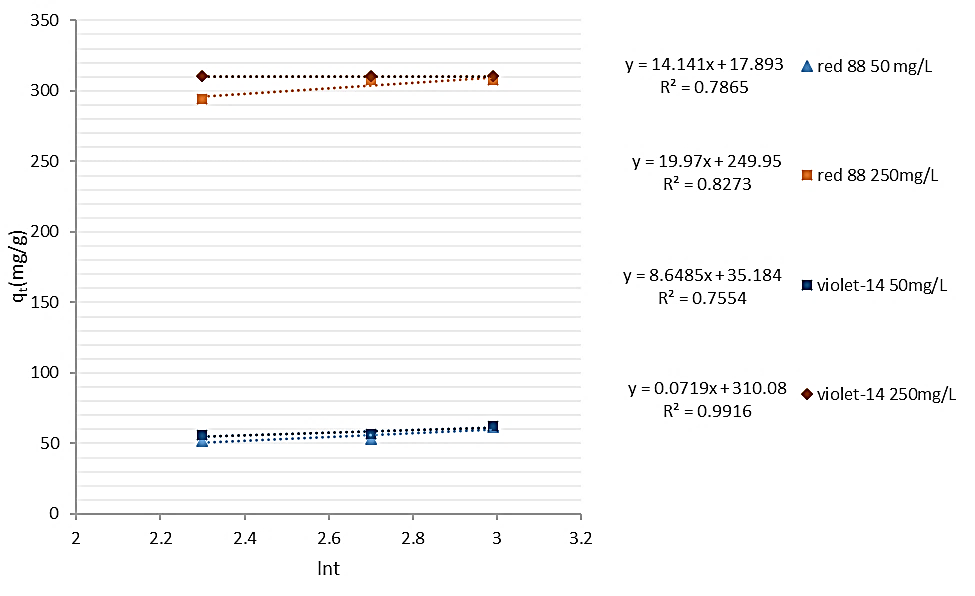


| Blue 54 50 | | Blue54 250 | | Congo red 50 | | Congo red250 | |
| --- | --- | --- | --- | --- | --- | --- | --- |
| 2.3 | 57.465 | 2.3 | 272.16 | 2.3 | 61.53 | 2.3 | 311.53 |
| 2.7 | 59.125 | 2.7 | 277.75 | 2.7 | 62.29 | 2.7 | 311.95 |
| 2.99 | 59.375 | 2.99 | 281.25 | 2.99 | 62.41 | 2.99 | 312 |


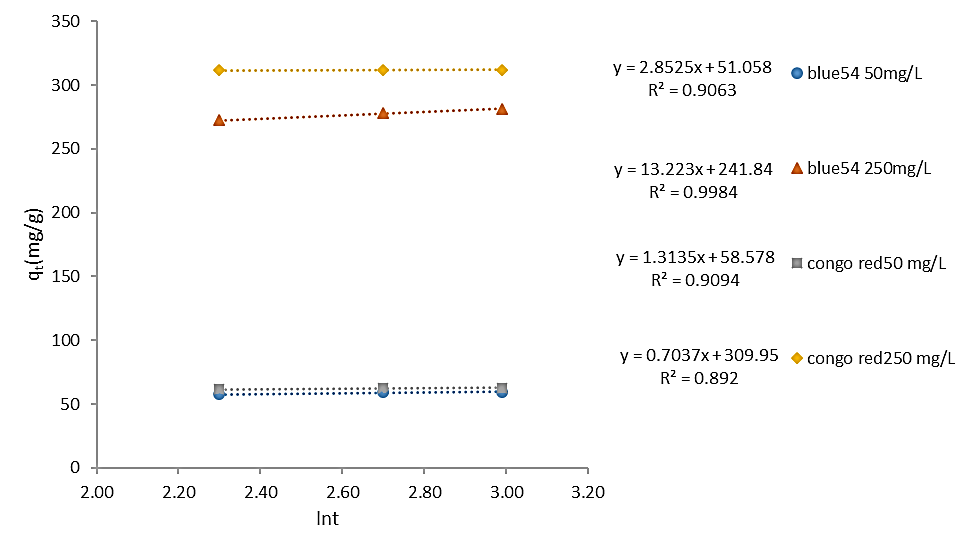


Kinetics plot for the adsorption of dyes by ZPBIF-1. The Elovich model (Contact time=20 min, Tem=25-65°C, Adsorbent mass=0. 08g/L, Initial concertation 50 and 250mg/L, Stirring=400 rpm and pH=2).

**Table 3** Kinetics constants for the adsorption of dyes by ZPBIF-1 (Contact time=20 min, Tem=25-65°C,

Adsorbent mass=0. 08g/L, Initial concertation 50 and 250mg/L, Stirring=400 rpm and pH=2).

|  |  | Acid Red 88 | | Basic Violet 14 | | Basic blue 54 | | Congo red | |
| --- | --- | --- | --- | --- | --- | --- | --- | --- | --- |
|  | C_0_ (mg/L) | 50 | 250 | 50 | 250 | 50 | 250 | 50 | 250 |
| Pseudo – first – order model | q_e,exp_(mg/g) | 61.87 | 307.55 | 62.18 | 310.30 | 59.37 | 281.25 | 62.41 | 311.87 |
|  | q_e,cal_ (mg/g) | 55.63 | 370.59 | 53.57 | 183.71 | 61.90 | 252.29 | 59.24 | 270.20 |
|  | K_1_ (min^-1^) | 0.1376 | 0.3894 | 0.1704 | 0.6632 | 0.3614 | 0.2998 | 0.4054 | 0.5918 |
|  | R^2^ | 0.9333 | 0.9732 | 0.9160 | 0.9304 | 0.9983 | 0.9845 | 0.9981 | 0.9930 |
| first – order model | C_0_ | 64.80 | 185.04 | 61.75 | 107.66 | 34.80 | 183.60 | 36.88 | 102.53 |
|  | K_1_ | 0.2066 | 0.2208 | 0.2384 | 0.2548 | 0.1554 | 0.1185 | 0.3401 | 0.3329 |
|  | R^2^ | 0.8630 | 0.9301 | 0.888 | 0.7745 | 0.8722 | 0.8470 | 0.9774 | 0.8386 |
| Pseudo – second – order model | q_e,cal_ (mg/g) | 59.88 | 312.5 | 60.97 | 312.5 | 58.82 | 277.77 | 62.50 | 312.5 |
|  | K_2_(g/min.mg) | 0.0228 | 0.0256 | 0.0389 | 0 | 0.0741 | 0.0648 | 0.3657 | 1.024 |
|  | R^2^ | 0.9798 | 0.9994 | 0.9926 | 1 | 0.9979 | 0.9999 | 1 | 1 |
| second – order model | C_0_ | 2.94 | 89.28 | 1.44 | 10.39 | 28.01 | 142.85 | 0.42 | 28.32 |
|  | K_2_ | 0.0806 | 0.0138 | 0.1575 | 0.029 | 0.0199 | 0.0018 | 0.7185 | 0.1314 |
|  | R^2^ | 0.5178 | 0.8610 | 0.5106 | 0.7872 | 0.9682 | 0.9310 | 0.7514 | 0.9737 |
| Intra-particle diffusion  model | K_p_(g/min.mg) | 7.6325 | 10.3320 | 4.6708 | 0.0381 | 1.4859 | 6.9672 | 0.6844 | 0.3658 |
|  | C (mg/g) | 26.29 | 263.53 | 40.30 | 310.13 | 52.95 | 250.35 | 59.45 | 310.42 |
|  | R^2^ | 0.8209 | 0.7934 | 0.7896 | 0.9977 | 0.8812 | 0.9930 | 0.8846 | 0.8637 |
| Elovich constants | R^2^ | 0.7865 | 0.8273 | 0.7554 | 0.9916 | 0.9063 | 0.9984 | 0.9094 | 0.892 |
|  | α (mg/g.min) | 50.6321840032 | 5454780.13  12788 | 508.2847556925 | ∞ | 169642386.81368 | 1169223763.5818 | 3.06752219E+190 | 1.36661039E+191 |
|  | ß (g/mg) | 0.070 | 0.050 | 0.115 | 13.90 | 0.350 | 0.075 | 0.761 | 1.421 |

| red 88 | | Violet-14 | | Blue 54 | | Congo red | |
| --- | --- | --- | --- | --- | --- | --- | --- |
| 0.0033 | 3.41 | 0.0033 | 5.516 | 0.0033 | 3.16 | 0.0033 | 6.43 |
| 0.0031 | 4.11 | 0.0031 | 4.16 | 0.0031 | 3.02 | 0.0031 | 2.0521 |
| 0.0029 | 4.81 | 0.0029 | 3.83 | 0.0029 | 2.6910 | 0.0029 | 2.035 |


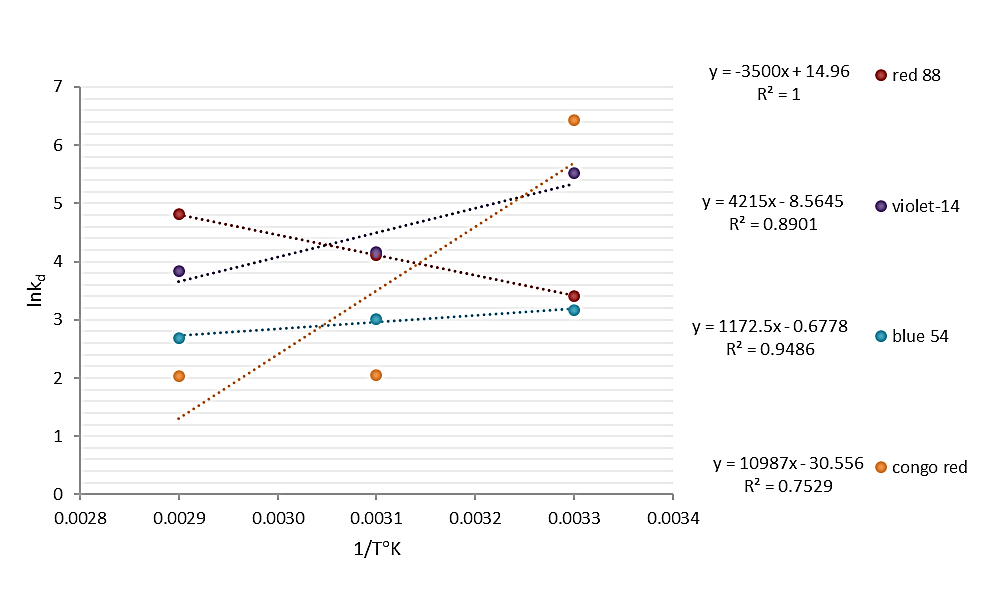


**Table 4** Thermodynamic parameters for the adsorption of dyes on ZPBIF-1

| ΔS°(J/mol.K) | ΔH°(Kj/mol) | ΔG°(Kj/mol) | (k) | (Acid red88) (PPM) |
| --- | --- | --- | --- | --- |
| 124.31 | 29.08 | -8.44 | 298 | 100/mg/L |
|  |  | -10.86 | 318 |  |
|  |  | -13.51 | 338 |  |
| ΔS°(J/mol.K) | ΔH°(Kj/mol) | ΔG°(Kj/mol) | (k) | (Basic Violet 14) (PPM) |
| -71.17 | -35.02 | -13.65 | 298 | 100 mg/L |
|  |  | -10.99 | 318 |  |
|  |  | -10.75 | 338 |  |
| ΔS°(J/mol.K) | ΔH°(Kj/mol) | ΔG°(Kj/mol) | (k) | (Basic Blue54) (PPM) |
| -5.63 | -9.74 | -7.82 | 298 | 100 mg/L |
|  |  | -7.98 | 318 |  |
|  |  | -7.55 | 338 |  |
| ΔS°(J/mol.K) | ΔH°(Kj/mol) | ΔG°(Kj/mol) | (k) | (Congo red) (PPM) |
| -253.92 | -91.30 | -15.92 | 298 | 100 mg/L |
|  |  | -5.42 | 318 |  |
|  |  | -5.71 | 338 |  |
|  |  |  |  |  |

**Table 5:** Removal of Acid Red 88 dye from textile wastewater sample.

| dye | Initial concentration  (mg/L) | Final concentration (mg/L) | % Removal |
| --- | --- | --- | --- |
| Acid Red 88 | 50 | 3.55 | 92.9 |

y=0.013 x+0.037=0

x=2.84

2.84×25 cc÷20cc

=3.55

50-3.55÷50=0.929 ×100=92.9
