# Supplementary material for: Design, synthesis, and characterization of a novel Zn(II)-2-phenyl benzimidazole framework for the removal of organic dyes
Source: Sci Rep. 2022 Jul 20;12:12431. doi: 10.1038/s41598-022-16753-8 (PMC9300708; doi:10.1038/s41598-022-16753-8)
Supplement: Supplementary file 5 — Supplementary Information 5. [file 41598_2022_16753_MOESM5_ESM.docx]

**Novel Zn (II)-2-phenyl benzimidazole framework (ZPBIF-1) for the efficient removal of organic dyes: synthesis, characterization, adsorption, kinetic, and thermodynamic studies**

Shabnam Alibakhshi^1^, Ashraf S. Shahvelayati^a1^, Shabnam Sheshmani^1^, Maryam Ranjbar^2^, Saeid Souzangarzadeh^1^

*^1^Department of Chemistry, College of Basic Sciences, Yadegar-e- Imam Khomeini (RAH) Shahre Ray Branch, Islamic Azad University, Tehran, Iran.* * Corresponding Author: [avelayati@yahoo.com](mailto:avelayati@yahoo.com), [a_shahvelayati@iausr.ac.ir](mailto:a_shahvelayati@iausr.ac.ir)

*^2^Department of Chemical Technologies, Iranian Research Organization for Science and Technology (IROST), Tehran, Iran*

**Supplementry file 5: The molecular structures of dyes and IR, ^1^HNMR, ^13^CNMR and the mass spectra of ZPBIF-1.**

**The molecular structures of dyes.**

Chemical structure of Acid red 88 Chemical structure of Congo red

Chemical structure of Basic Blue 54 Chemical structure of Basic Violet -14

**The FT-IR spectrum of Zn(II)-2-phenyl benzimidazole framework.**

Wavenumber cm^-1^

**^1^H NMR ZPBIF-1**

**^13^ C NMR ZPBIF-1**

**
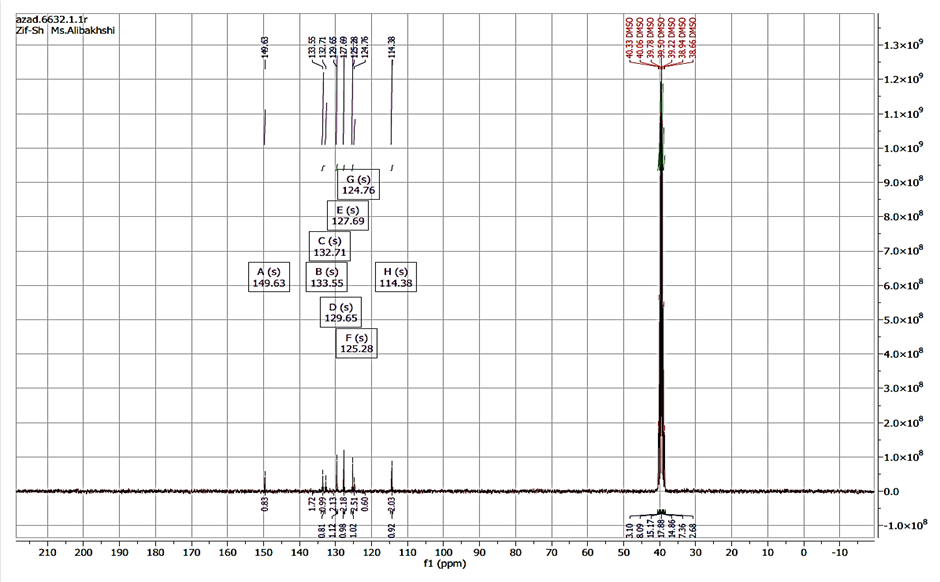
**

**The mass spectra of ZPBIF-1**

**
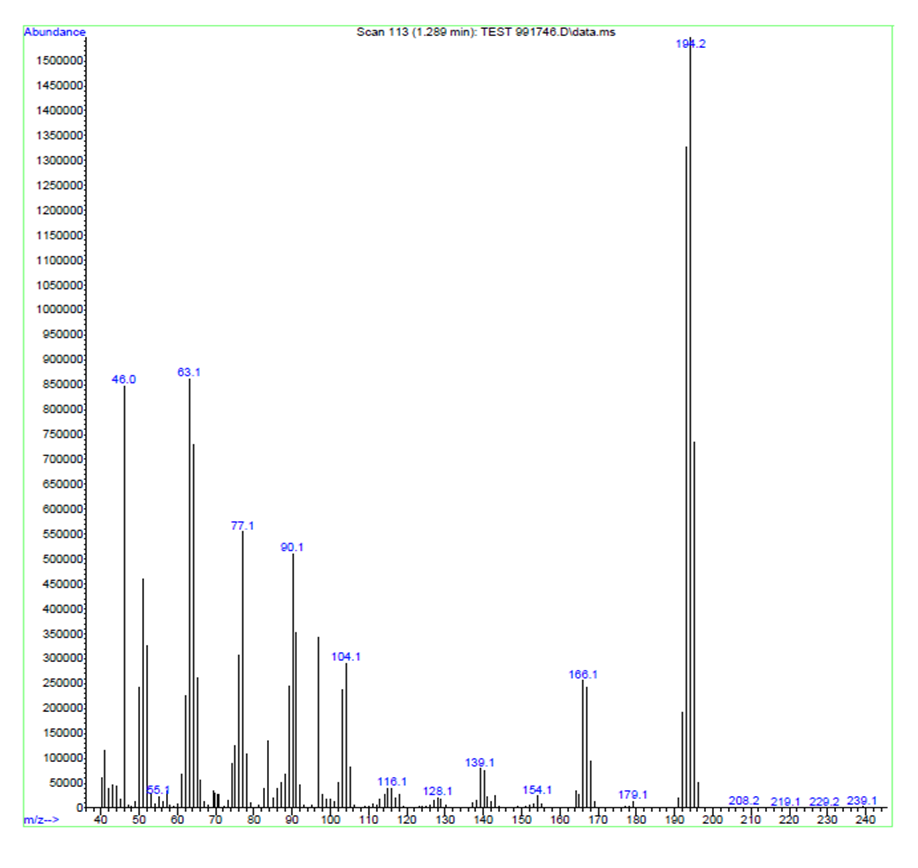
**
