# Supplementary material for: Design, synthesis, and characterization of a novel Zn(II)-2-phenyl benzimidazole framework for the removal of organic dyes
Source: Sci Rep. 2022 Jul 20;12:12431. doi: 10.1038/s41598-022-16753-8 (PMC9300708; doi:10.1038/s41598-022-16753-8)
Supplement: Supplementary file 6 — Supplementary Information 6. [file 41598_2022_16753_MOESM6_ESM.docx]

**Novel Zn (II)-2-phenyl benzimidazole framework for the efficient removal of organic dyes: synthesis, characterization, adsorption, kinetic, and thermodynamic studies**

Shabnam Alibakhshi^1^, Ashraf S. Shahvelayati^a1^, Shabnam Sheshmani^1^, Maryam Ranjbar^2^, Saeid Souzangarzadeh^1^

*^1^Department of Chemistry, College of Basic Sciences, Yadegar-e- Imam Khomeini (RAH) Shahre Ray Branch, Islamic Azad University, Tehran, Iran.* * Corresponding Author: [avelayati@yahoo.com](mailto:avelayati@yahoo.com), [a_shahvelayati@iausr.ac.ir](mailto:a_shahvelayati@iausr.ac.ir)

*^2^Department of Chemical Technologies, Iranian Research Organization for Science and Technology (IROST), Tehran, Iran*

**Supplementary file 6: Nitrogen adsorption/desorption isotherm of ZPBIF-1**

| Starting point | 1 |  |
| --- | --- | --- |
| End point | 22 |  |
| Slope(Linear) | 0.01174 |  |
| Intercept(Linear) | 33.086 |  |
| Correlation coefficient | 0.053411 |  |
| Vm | 85.176 | [cm^3^(STP) g^-1^] |
| a_s,Lang_ | 370.73 | [m^2^ g^-1^] |
| B | 0.00035484 |  |

| No | *p*/kPa | *p*/*V_a_* |
| --- | --- | --- |
| 1 | 1.2965 | 20.353 |
| 2 | 5.4717 | 26.499 |
| 3 | 7.543 | 33.793 |
| 4 | 10.507 | 27.234 |
| 5 | 13.485 | 40.985 |
| 6 | 16.116 | 39.768 |
| 7 | 19.577 | 43.101 |
| 8 | 23.75 | 36.765 |
| 9 | 26.295 | 31.357 |
| 10 | 30.673 | 30.208 |
| 11 | 34.864 | 31.523 |
| 12 | 39.259 | 34.641 |
| 13 | 43.457 | 35.228 |
| 14 | 47.758 | 34.617 |
| 15 | 51.939 | 37.028 |
| 16 | 56.322 | 35.828 |
| 17 | 60.522 | 38.846 |
| 18 | 69.016 | 38.389 |
| 19 | 73.409 | 36.666 |
| 20 | 77.613 | 33.736 |
| 21 | 81.861 | 30.203 |
| 22 | 85.148 | 21.412 |
